# Poly(A) polymerase is required for RyhB sRNA stability and function in *Escherichia coli*

**DOI:** 10.1261/rna.067181.118

**Published:** 2018-11

**Authors:** Dhriti Sinha, Lisa M. Matz, Todd A. Cameron, Nicholas R. De Lay

**Affiliations:** 1 Department of Microbiology and Molecular Genetics, McGovern Medical School, University of Texas Health Science Center, Houston, Texas 77030, USA; 2 MD Anderson Cancer Center UTHealth Graduate School of Biomedical Sciences, University of Texas Health Science Center, Houston, Texas 77030, USA

**Keywords:** poly(A) polymerase, *pcnB*, Hfq, small RNAs, RNase E

## Abstract

Small regulatory RNAs (sRNAs) are an important class of bacterial post-transcriptional regulators that control numerous physiological processes, including stress responses. In Gram-negative bacteria including *Escherichia coli*, the RNA chaperone Hfq binds many sRNAs and facilitates pairing to target transcripts, resulting in changes in mRNA transcription, translation, or stability. Here, we report that poly(A) polymerase (PAP I), which promotes RNA degradation by exoribonucleases through the addition of poly(A) tails, has a crucial role in the regulation of gene expression by Hfq-dependent sRNAs. Specifically, we show that deletion of *pcnB*, encoding PAP I, paradoxically resulted in an increased turnover of certain Hfq-dependent sRNAs, including RyhB. RyhB instability in the *pcnB* deletion strain was suppressed by mutations in *hfq* or *ryhB* that disrupt pairing of RyhB with target RNAs, by mutations in the 3′ external transcribed spacer of the *glyW-cysT-leuZ* transcript (3′ETS^LeuZ^) involved in pairing with RyhB, or an internal deletion in *rne*, which encodes the endoribonuclease RNase E. Finally, the reduced stability of RyhB in the *pcnB* deletion strain resulted in impaired regulation of some of its target mRNAs, specifically *sodB* and *sdhCDAB.* Altogether our data support a model where PAP I plays a critical role in ensuring the efficient decay of the 3′ETS^LeuZ^. In the absence of PAP I, the 3′ETS^LeuZ^ transcripts accumulate, bind Hfq, and pair with RyhB, resulting in its depletion via RNase E-mediated decay. This ultimately leads to a defect in RyhB function in a PAP I deficient strain.

## INTRODUCTION

Small regulatory RNAs (sRNAs) are transcripts ranging in size from 50 to 300 nucleotides (nt) that have been shown to regulate nearly every aspect of bacterial behavior and physiology including virulence (Toledo-Arana et al. 2009; Gripenland et al. 2010; Felden et al. 2011; Koo et al. 2011; Bardill and Hammer 2012; Hébrard et al. 2012; Mann et al. 2012; Rutherford and Bassler 2012; Caldelari et al. 2013; Holmqvist and Wagner 2017), biofilm development (Thomason et al. 2012; Jørgensen et al. 2013; Zhao et al. 2013; Parker et al. 2017), antibiotic resistance (Parker and Gottesman 2016; Zhang et al. 2017; Felden and Cattoir 2018), and metabolism (Durand and Storz 2010; Gimpel et al. 2010; Richards and Vanderpool 2011; Salvail and Massé 2012; Bobrovskyy and Vanderpool 2013; McClure et al. 2013; Mandin et al. 2016; Pannuri et al. 2016; Gonzalez et al. 2017). Many sRNAs regulate these processes by recognizing and binding specific target mRNAs through base-pairing resulting in changes in mRNA transcription, translation, or stability depending on the nature of this interaction. For example, sRNAs can inhibit translation by base-pairing with the mRNA translation initiation region directly blocking ribosome access to the Shine–Delgarno sequence (Møller et al. 2002a). Alternatively, negative regulation of gene expression can also be achieved by sRNA-induced RNase E mediated decay of mRNAs (Pfeiffer et al. 2009; Bandyra et al. 2012). Furthermore, sRNAs can positively regulate gene expression by base-pairing with a mRNA as it is being transcribed, preventing intramolecular base-pairing in the 5′ untranslated region (5′-UTR) that would otherwise lead to transcription attenuation (Sedlyarova et al. 2016).

In *Escherichia coli* and other Gram-negative bacteria, many sRNAs encoded in *trans* require the RNA-binding protein chaperone Hfq to function (Waters and Storz 2009; Storz et al. 2011). Hfq stabilizes sRNAs by binding and occluding an RNase E cleavage site (Moll et al. 2003), but also serves as a matchmaker by facilitating annealing between sRNAs and their target mRNAs (Møller et al. 2002b; Zhang et al. 2002; Lease and Woodson 2004; Soper and Woodson 2008). Although Hfq has been studied extensively, recent research has identified PNPase, encoding the 3′ → 5′ exoribonuclease polynucleotide phosphorylase, as another mediator of sRNA stability and function (De Lay and Gottesman 2011). PNPase degrades at least some sRNAs not associated with Hfq (Viegas et al. 2007; Andrade et al. 2012). However, PNPase binds and stabilizes many Hfq-dependent sRNAs (Bandyra et al. 2016) and has been further shown to impact sRNA–mRNA pairing (Cameron and De Lay 2016).

The crucial role of PNPase in modulating sRNA stability and function was discovered in a combined genetic selection and screen designed to isolate mutants defective for sRNA function in *E. coli*. Loss-of-function point mutations identified in *pnp* interfered with target gene regulation by Hfq-dependent sRNAs including RyhB (De Lay and Gottesman 2011). RyhB is one of the best-characterized sRNAs in *E. coli*. Under iron replete conditions, RyhB expression is repressed by the iron bound form of the iron sensing transcriptional regulator protein Fur (ferric uptake regulator). Under iron limiting conditions Fur can no longer associate with free Fe^2+^ efficiently and consequently with DNA, which leads to RyhB expression. RyhB, in turn, acts to modulate iron homeostasis by down-regulating the expression of a large number of mRNAs encoding nonessential iron-containing proteins including *sdhCDAB* and *sodB,* which encode succinate dehydrogenase and superoxide dismutase, respectively (Massé and Gottesman 2002; Massé et al. 2003, 2005, 2007; Richards and Vanderpool 2011).

In the same genetic selection and screen that isolated *hfq* and *pnp* mutants, independent point mutants were obtained in *pcnB*, the gene encoding poly(A) polymerase (PAP I), which were not investigated in detail (De Lay and Gottesman 2011). PAP I catalyzes polyadenylation of the 3′ extremities of RNA substrates and has a preference for Rho-independent transcription terminators containing 2–6 nt single-stranded extensions (Mohanty and Kushner 2008, 2011, 2016; Régnier and Hajnsdorf 2013). Poly(A) tails promote transcript decay by providing toe-holds for 3′ → 5′ exoribonucleases like RNase II, RNase R, and PNPase. PAP I interacts with Hfq and PNPase to form a polyadenylation complex. Hfq has been shown to stimulate polyadenylation of mRNAs by PAP I, most likely by binding to and recruiting PAP I to the 3′ ends of RNA substrates (Hajnsdorf and Régnier 2000; Le Derout et al. 2003; Mohanty et al. 2004).

PAP I adds poly(A) tails to many different classes of cellular RNAs (mRNAs, rRNAs, tRNAs, sRNAs, viral RNAs) (Régnier and Hajnsdorf 2013; Mohanty and Kushner 2016), and while the majority of *E. coli* ORFs undergo polyadenylation under exponential growth conditions, only a small fraction of them are polyadenylated at a specific time (Mohanty and Kushner 2006). Many sRNAs that do not require Hfq for stability and function have been shown to be polyadenylated in vivo, e.g., RNA I, Sok, Oop, SraL, SraG, and GlmY, and are subsequently degraded by exoribonucleases (Régnier and Hajnsdorf 2013; Ruiz-Larrabeiti et al. 2016). Interestingly, previous data have shown that sRNAs that require Hfq for their stability, e.g., MicA and RybB, can also be targeted for degradation by PNPase and PAP I, but only when these sRNAs are not bound by Hfq (Andrade and Arraiano 2008; Andrade et al. 2012; Cameron and De Lay 2016).

In this study, we have further investigated the possible mechanisms by which the PAP I mediated polyadenylation led to a defect in sRNA function. Here, we report that deletion of *pcnB* encoding PAP I resulted in a significant reduction in RyhB stability and consequently a defect in RyhB-mediated repression of *sdhCDAB* and *sodB* transcripts. We provide evidence that the increased turnover of RyhB in a *pcnB* deletion strain is due to increased accumulation of the 3′ETS^LeuZ^, which promotes more rapid RyhB degradation by RNase E as a consequence of base-pairing interactions with this sRNA. Finally, we show that PAP I can stabilize another Hfq-dependent sRNA, MicA, but not others (GcvB, CyaR, ChiX, and MgrR), suggesting a specialized role of PAP I in conferring stability to a specific subset of Hfq-dependent sRNAs. This work provides further insight into how yet another protein previously known to be involved in initiating RNA decay contributes to sRNA-dependent gene regulation.

## RESULTS

### Poly(A) polymerase stabilizes RyhB

**FIGURE 1.**
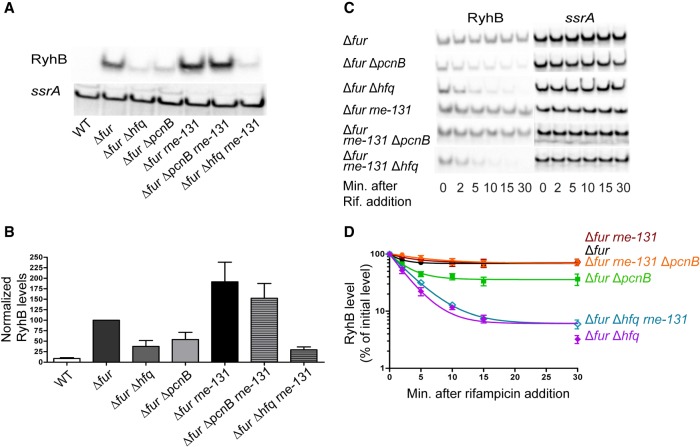
RyhB is rapidly degraded in the absence of poly(A) polymerase in an RNase E-dependent mechanism. (*A*,*B*) Northern blot analysis to assess RyhB steady-state levels. Overnight cultures of strain NRD1138 (WT), an isogenic Δ*fur* strain (DS024), or derivatives of this Δ*fur* strain harboring deletions in *hfq* (DS027), *pcnB* (DS025), *rne* (DS069), *rne* and *pcnB* (DS082), and *rne* and *hfq* (DS153) were diluted 200-fold in fresh MOPS EZ rich defined media supplemented with 0.4% glycerol. All cultures were subsequently grown to late exponential phase (OD_600_ of 1.0), and samples were collected for RNA extraction. (*C*,*D*) RNA stability time course experiment to determine the intrinsic stability of RyhB. Briefly, overnight cultures of the Δ*fur* parent (DS024) and its derived mutant strains (Δ*fur* Δ*pcnB*, DS025; Δ*fur* Δ*hfq*, DS027; Δ*fur rne-131*, DS069; Δ*fur rne-131* Δ*pcnB*, DS082; Δ*fur rne-131* Δ*hfq*, DS153) were grown to OD_600_ of 1.0 as described above and a culture sample was taken. Rifampicin was added to each culture to stop total transcription, and additional culture samples were taken 2, 5, 10, 15, and 30 min after rifampicin addition. All samples were subjected to RNA extraction and were prepared for northern blot analysis as described in Materials and Methods. Representative northern blots are shown in *A* and *C*. (*B*,*D*) RyhB signal intensities in the northern blots were quantified and normalized to their corresponding loading controls (*ssrA*). sRNA decay curves were generated by fitting the normalized signal intensities for each time point. Points and error bars in the curves represent the means and the standard errors (SEM) of at least three independent experiments. RyhB half-life measurements corresponding to RNA stability curves (*D*) are listed in Table 1.

In a previous study (De Lay and Gottesman 2011), strains harboring null mutations in *hfq*, *pnp*, or *pcnB* encoding the RNA chaperone Hfq, the exoribonuclease PNPase, or the poly(A) polymerase PAP I, respectively, were recovered in a genetic selection and screen designed to isolate mutants defective for sRNA-mediated gene regulation. The selection was for mutations that allowed an *E. coli* Δ*fur* strain to grow on minimal succinate medium. In a *fur* deletion strain, RyhB is constitutively expressed and accumulates under the protection of Hfq. In turn, Hfq promotes base-pairing with the *sdhCDAB* mRNA, blocking expression of succinate dehydrogenase complex and consequently leading to an inability of a *fur* mutant to grow on succinate as the sole carbon source (Massé and Gottesman 2002). Inactivation of *pnp* or *hfq* caused rapid turnover of RyhB leading to up-regulation of the *sdhCDAB* transcript, and consequently allowed a Δ*fur* stain to grow on succinate as the sole carbon source (De Lay and Gottesman 2011). Since the point mutations in *pcnB* allowed growth on succinate minimal medium, we tested the effect of a *pcnB* deletion on RyhB stability. To test this, we first examined RyhB steady-state levels in exponentially growing cultures of Δ*fur*, Δ*fur* Δ*pcnB*, and Δ*fur* Δ*hfq* strains. Introduction of a *pcnB* deletion into the Δ*fur* strain resulted in a decrease in RyhB steady-state levels by 40% to 50% and were comparable to that in a Δ*fur* Δ*hfq* strain (Fig. 1A,B). As expected, RyhB was not detectable in a wild-type *fur*^+^ strain (WT). Next, we determined RyhB stability in exponentially growing cultures of the aforementioned strains by blocking transcription initiation by adding rifampicin. RyhB levels were monitored by northern blot analysis of samples taken after transcription inhibition. RyhB was very stable in the Δ*fur* parent background under our experimental conditions, while introduction of an *hfq* deletion drastically reduced the stability of this sRNA (Table 1; Fig. 1C,D). Interestingly, introduction of a *pcnB* single deletion reduced RyhB stability significantly, but to a lesser extent than what was observed for an *hfq* mutant (Table 1; Fig. 1C,D).

**TABLE 1.**
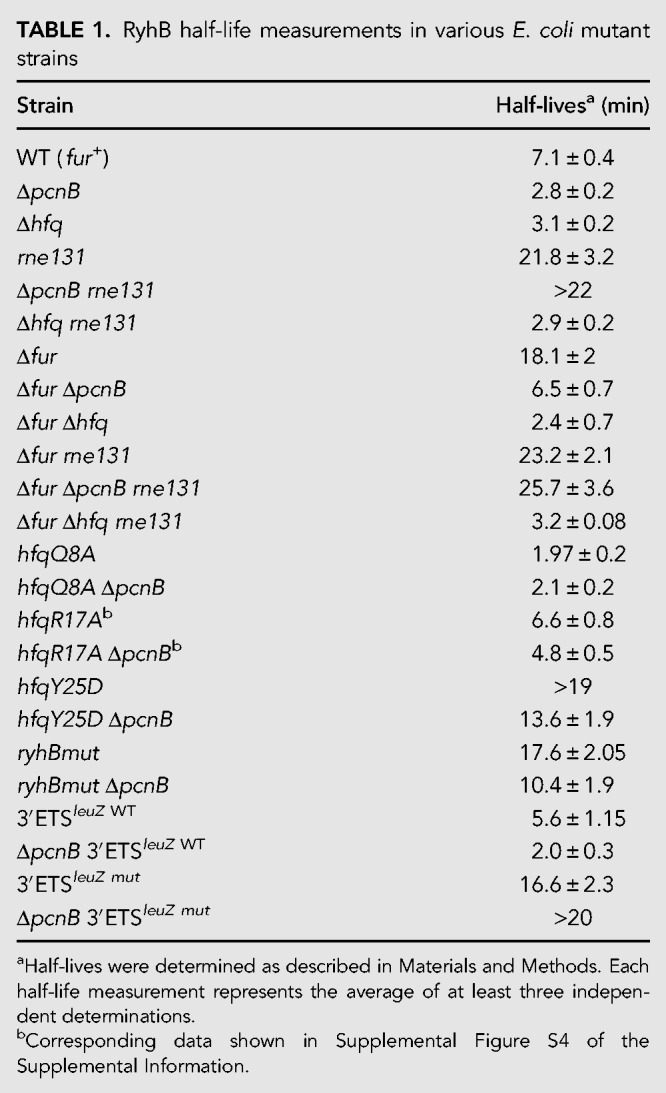
RyhB half-life measurements in various *E*. *coli* mutant strains

**FIGURE 2.**
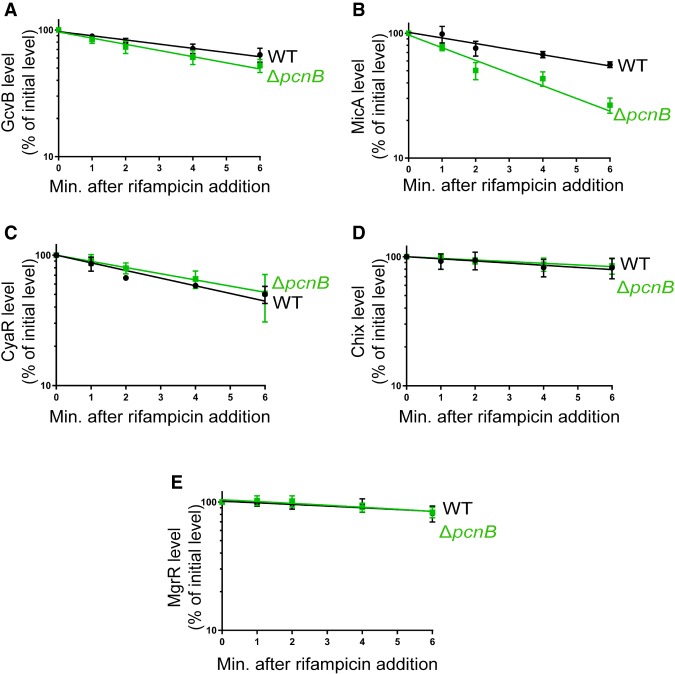
A subset of Hfq-dependent sRNAs are unstable in the absence of poly(A) polymerase. RNA stability time-course experiments to determine the intrinsic stabilities of GcvB (*A*), MicA (*B*), CyaR (*C*), ChiX (*D*), and MgrR (*E*) sRNAs. (*A*,*B*) Strain TC279 (WT; *fur*^+^), which has MicA under the control of the *ryhB* promoter, and an isogenic Δ*pcnB* strain (Δ*pcnB*, DS120) were grown to exponential phase. Dipyridyl was added to each culture for 15 min to induce MicA expression, a sample was taken, rifampicin was added to block transcription, and additional samples were taken 1, 2, 4, and 6 min after rifampicin addition. RNA was extracted and northern blot analysis was performed probing for MicA or GcvB as described in Materials and Methods. To determine intrinsic stabilities of CyaR (*C*), ChiX (*D*), and MgrR (*E*), the wild-type strain (NRD1138) and its derived *pcnB* mutant (NRD1198) were grown to exponential phase, CyaR was induced from its native promoter by cAMP addition, a sample was taken after 15 min of induction, rifampicin was then added, and additional samples were taken 1, 2, 4, and 6 min after transcription inhibition. Northern blot analysis was performed using RNA extracted from these samples probing for CyaR, ChiX, and MgrR. Representative northern blots for each sRNA are shown in Supplemental Figure S1 (Supplemental Information). For the decay curves, sRNA signal intensities from the northern blots were quantified and normalized to their corresponding loading controls (*ssrA* or 5S). Points and error bars in the curves represent the means and the standard errors (SEM) of at least three independent experiments.

Since deletion of *pcnB* led to increased turnover of RyhB, we decided to test whether PAP I was also required for the stability of other Hfq-dependent sRNAs including MicA, GcvB, CyaR, ChiX, and MgrR. We determined the stability of these sRNAs in exponentially growing cultures of a Δ*pcnB* and a wild-type strain as described above. Deletion of *pcnB* led to ∼50% reduction in MicA stability compared to the wild-type strain, while there were no significant differences in stability of GcvB, CyaR, ChiX, or MgrR between a wild-type and a Δ*pcnB* strain (Fig. 2; Supplemental Fig. S1). Overall, our data demonstrated that deletion *of pcnB* led to increased turnover of at least two Hfq-dependent sRNAs, RyhB and MicA.

### Poly(A) polymerase is important for RyhB-mediated target gene regulation

**FIGURE 3.**
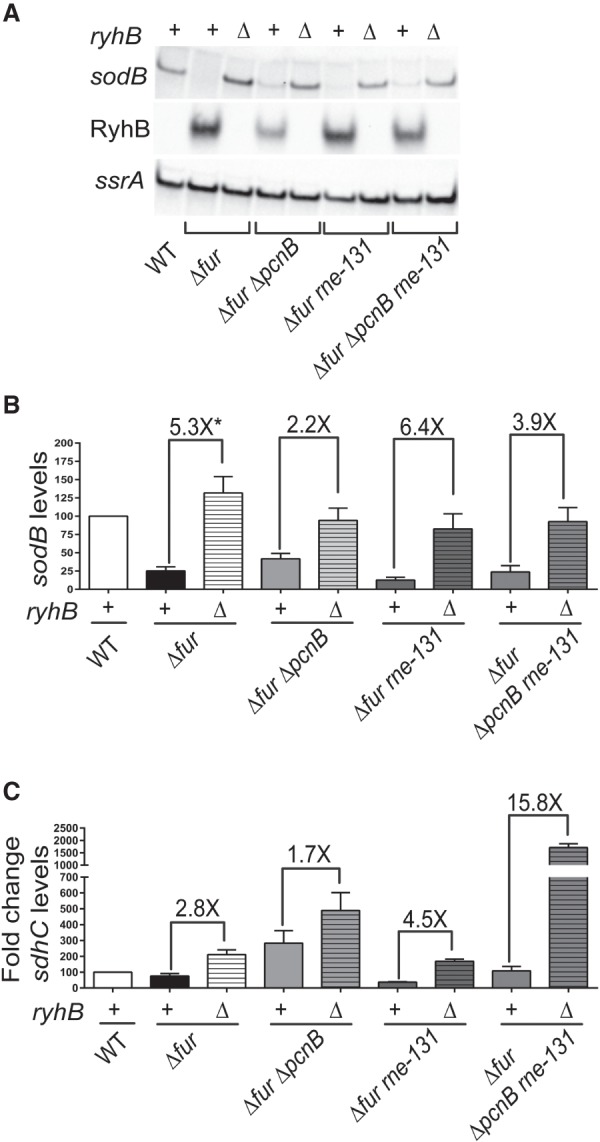
In the absence of poly(A) polymerase, RyhB does not efficiently regulate *sodB* and *sdhCDAB* target mRNAs. (*A*,*B*) Northern blot analysis was used to determine RyhB and *sodB* steady state in the wild-type and derived isogenic mutant strains, and (*C*) qRT-PCR analysis was used to determine *sdhC* levels. Wild-type parent (WT [*fur*^+^]; NRD1138) and its derived isogenic mutants (Δ*fur*, DS024; Δ*fur* Δ*pcnB*, DS025; Δ*fur rne-131*, DS069; Δ*fur* Δ*pcnB rne-131,* DS082; Δ*fur* Δ*ryhB*, NRD1546; Δ*fur* Δ*pcnB* Δ*ryhB*, NRD1547; Δ*fur rne-131* Δ*ryhB*, NRD1550; Δ*fur* Δ*pcnB rne-131* Δ*ryhB*, NRD1551) were diluted 200-fold in fresh MOPS EZ rich defined media supplemented with 0.4% glycerol and grown to late exponential phase (OD_600_ of 1.0), and samples for RNA extraction were collected. Representative northern blots are shown (*A*). (*B*,*C*) Graphs are presented that display the relative expression levels of *sodB* and *sdhC* mRNA in a wild-type strain and derived mutant strains. Briefly, the signal intensities for *sodB* and *sdhC* transcripts were first quantified from northern blots or qRT-PCRs. The signal intensity was then normalized to the *ssrA* transcript level, which served as the loading control, and subsequently the expression level relative to the *fur*^+^ (WT) strain NRD1138, which was set at 100%, was calculated. *sdhC* transcript fold changes relative to NRD1138 were calculated via the ΔΔC_t_ method. Asterisk (*) in *B* indicates that the calculated fold change is >5.3× since *sodB* steady-state determination in DS024 (Δ*fur*) strain was not accurate due to very low signal intensity. Data shown in *B* and *C* represent the mean (±SEM) of at least three independent experiments. Probes and primers used are listed in Supplemental Table S2.

Based on the results reported above, we investigated whether this defect in RyhB stability in a *pcnB* deletion mutant led to a subsequent defect in RyhB-dependent target gene regulation. To test this idea, we focused on the two well-characterized RyhB target mRNAs, *sodB* and *sdhCDAB*. We determined the relative levels of *sodB* using northern blotting and *sdhCDAB* by qRT-PCR using specific primers to probe for transcripts containing *sdhC* and *sdhD* in exponential phase cultures of a Δ*fur* strain or derived strain harboring a deletion in *pcnB* (Δ*pcnB*) in the presence (+*ryhB*) or absence (Δ*ryhB*) of *ryhB*. We observed a greater than fivefold down-regulation of *sodB* transcript levels by RyhB in the Δ*fur* strain (Fig. 3A,B; compare Δ*fur* versus Δ*fur* Δ*ryhB*). However, in the corresponding isogenic Δ*pcnB* strains, RyhB-dependent regulation of *sodB* was only 2.2-fold (Fig. 3A,B; compare Δ*fur* Δ*pcnB* versus Δ*fur* Δ*pcnB* Δ*ryhB*). Furthermore, we found that *sodB* was turned over less rapidly in a Δ*pcnB* mutant (*t*_1/2_ = 4.7 min) relative to a wild-type (*t*_1/2_ = 3.4 min) strain upon RyhB induction (Supplemental Fig. S2). The decreased rate of *sodB* target degradation in a *pcnB* mutant indicated a defect in RyhB-mediated *sodB* repression. Similarly, qRT-PCR analysis showed a nearly threefold down-regulation of *sdhC* transcript levels by RyhB in a Δ*fur* strain background (Fig. 3C; compare Δ*fur* versus Δ*fur* Δ*ryhB*), whereas in the Δ*fur* Δ*pcnB* strain RyhB expression caused only a 1.7-fold reduction in *sdhC* levels (Fig. 3C; compare Δ*fur* Δ*pcnB* versus Δ*fur* Δ*pcnB* Δ*ryhB*). These results suggested a defect in RyhB-mediated regulation of *sdhCDAB* in the absence of PAP I. Interestingly, *sdhC* transcript levels were higher in a Δ*fur* Δ*pcnB* Δ*ryhB* strain than in the Δ*fur* Δ*ryhB* strain (Fig. 3C), suggesting that PAP I-mediated *sdhCDAB* regulation was both RyhB-dependent and RyhB-independent. Similar results were also obtained when we probed the *sdhCDAB* transcript by qRT-PCR using primers specific for *sdhD* instead of *sdhC* (Supplemental Fig. S3); i.e., introduction of the *pcnB* deletion into a Δ*fur* parent background up-regulated *sdhD* steady-state levels by threefold (Supplemental Fig. S3). Altogether, our results indicated that deletion of *pcnB* resulted in increased *sodB* transcript levels as a consequence of reduced RyhB levels, whereas the higher levels of *sdhCDAB* mRNA in the absence of PAP I were likely due to reduced RyhB levels and loss of PAP I-mediated regulation of the *sdhCDAB* transcript that was RyhB-independent.

Finally, we investigated whether PAP I can similarly impact MicA-mediated target gene regulation. To test this, we determined steady-state levels of two MicA-targets *ompA* and *ompX*, which encode outer membrane proteins, in a *pcnB* deletion and wild-type strain under conditions where MicA expression was induced or uninduced. We observed a 4.2-fold and 2.6-fold down-regulation of *ompA* and *ompX* transcript levels, respectively, by MicA in the wild-type strain while in the corresponding isogenic Δ*pcnB* strain, there was only a modest loss in MicA-dependent regulation of *ompA* (3.3-fold) and *ompX* (2.2-fold) (Supplemental Fig. S4). Taken together, our data indicated that PAP I plays a crucial role in promoting stability and function of RyhB and MicA.

### Hfq mediates accelerated decay of RyhB in the absence of poly(A) polymerase

**FIGURE 4.**
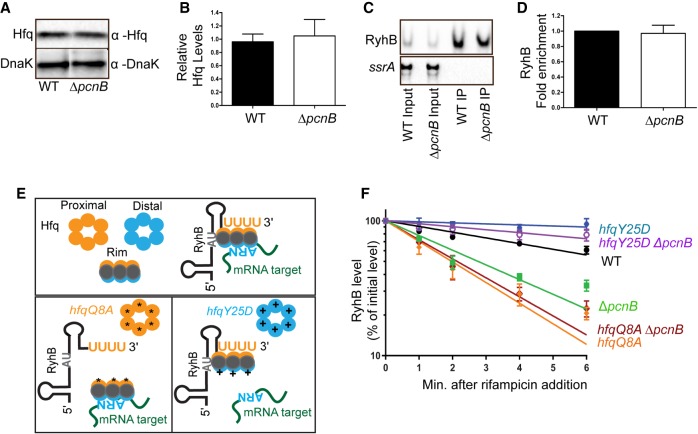
The accelerated decay of RyhB in the absence of poly(A) polymerase is mediated by Hfq. (*A*,*B*) Western blot analysis to determine Hfq protein levels. Samples were prepared for western blotting from exponential phase cultures of a *fur*^+^ strain (DS090), and its derived strain Δ*pcnB* (DS092) with anti-Hfq antibody. Protein band intensities were normalized to DnaK detected with an anti-DnaK antibody (Materials and Methods). Representative western blots are shown in *A*. Quantification of Hfq levels from those western blots normalized to DnaK levels are shown in *B*. (*C*,*D*) Coimmunoprecipitation of RyhB with Hfq. RyhB expression was induced in exponential cultures of a wild-type (NRD1138) and an isogenic Δ*pcnB* mutant (NRD1198). Hfq was immunoprecipitated with anti-Hfq antibody bound to protein-A-sepharose. RNA extracted from the input and elution fractions were loaded in 1:8 ratio, and RyhB and *ssrA* levels were determined via northern blot analysis (*C*). Fold enrichment of RyhB (*D*) was determined after quantification of the RyhB and *ssrA* signal intensities in those northern blots as described in Materials and Methods. (*E*) Schematic showing interactions between RyhB, mRNA targets, and Hfq based on work by Schu and coworkers (Zhang et al. 2013; Schu et al. 2015); a Q8A substitution in the proximal face of Hfq was shown to disrupt RyhB binding, whereas a Y25D substitution in the distal face of Hfq reduced binding of RyhB target mRNAs such as *sodB*. (*F*) RNA half-life experiments to determine RyhB stability in the wild-type strain and its derived isogenic *hfq* and *pcnB* mutants. Overnight cultures of the wild-type strain (WT [*fur*^+^]; NRD1138) and its derived mutants (Δ*pcnB*, NRD1198; *hfqQ8A*, DS060; *hfqY25D*, NRD1410; *hfqQ8A* Δ*pcnB*, DS072; *hfqY25D* Δ*pcnB,* DS185) were diluted 200-fold in fresh LB media. Cultures were subsequently grown to exponential phase, dipyridyl was added to induce RyhB expression, and a culture sample was taken after 15 min of induction. Rifampicin was added to each culture to stop total transcription, and additional culture samples were taken 1, 2, 4, and 6 min after rifampicin addition. RNA extraction and northern blot analysis were performed as described above. Representative northern blots are shown in Supplemental Figure S5 of Supplemental Information. For decay curves, RyhB signal intensities from the northern blots were quantified and normalized to their corresponding loading controls (SsrA). Data shown in *B*, *D*, and *F* represent the means and the standard errors (SEM) of at least three independent experiments. RyhB half-life measurements corresponding to RNA stability curves are listed in Table 1.

Based on our observations, we next investigated the mechanism by which PAP I promoted sRNA stability by focusing on RyhB. Since Hfq was essential for RyhB stability, we first determined whether PAP I impacted Hfq protein levels. Hfq protein levels were comparable between a wild-type and *pcnB* deletion strain under exponential growth conditions, indicating that the decreased stability of RyhB in a *pcnB* mutant strain was not due to lower levels of Hfq (Fig. 4A,B). Results from previous studies indicated that PAP I together with Hfq mediates efficient polyadenylation at the 3′ ends of Rho-independent terminators which subsequently facilitates Hfq binding to those mRNAs (Hajnsdorf and Régnier 2000; Mohanty and Kushner 2008, 2016; Mohanty et al. 2004; Régnier and Hajnsdorf 2013). We extended this idea to sRNAs and thus tested whether PAP I stabilized RyhB by increasing its binding to Hfq. We compared the total amount of RyhB to the amount immunoprecipitated with Hfq from cell lysates of a wild-type or an isogenic Δ*pcnB* strain; no significant difference was observed in the amount of RyhB that coimmunoprecipitated with Hfq between the wild-type and Δ*pcnB* strain (Fig. 4C,D). Altogether, our results indicated that PAP I did not facilitate RyhB binding to Hfq in vivo.

Recent studies directed toward understanding Hfq-RNA interactions have led to the identification of four distinct surfaces on Hfq: the proximal face, distal face, rim, and the C-terminal tail, each possessing unique structural characteristics which promote binding of different RNAs in particular configurations (Vogel and Luisi 2011; Zhang et al. 2013; Schu et al. 2015; Updegrove et al. 2016). sRNAs are classified into two distinct classes, Class I and Class II, based on their mode of Hfq binding. RyhB is a Class I Hfq-binding sRNA shown to bind to sites on the Hfq proximal face via the poly(U) tail of its Rho-independent terminator and the rim via its UA-rich sequence, while RyhB target mRNAs typically interact with the Hfq distal face via repeats of an ARN sequence motif (Fig. 4E). Binding studies with several Hfq mutants have demonstrated that specific conserved residues on the proximal face (Q8, F42, K56) and rim (R16, R17, R19) were important for binding of Class I sRNAs to Hfq, and substitutions in these residues negatively impacted sRNA steady-state levels and stability. On the other hand, specific residues on the Hfq distal face (Y25, I30) were important for cognate mRNA target binding, and substitutions in these residues stabilized Class I sRNAs by protecting them from the subsequent degradation following sRNA–mRNA pairing mediated by Hfq (Zhang et al. 2013; Schu et al. 2015; Updegrove et al. 2016).

Our results so far indicated that PAP I did not impact RyhB binding to Hfq, but at the same time was required to stabilize RyhB in vivo. This observation prompted us to determine whether PAP I was contributing to RyhB stability by impacting the interaction of RyhB target RNAs with Hfq. To test this possibility, we introduced different *hfq* point mutations (*hfqQ8A* [proximal face], *hfqR17A* [rim], and *hfqY25D* [distal face]) into a wild-type or a Δ*pcnB* mutant strain and monitored RyhB stability as described above (Fig. 4E,F; Supplemental Fig. S5). Introduction of the *hfqY25D* mutation not only suppressed the defect in RyhB stability observed for a Δ*pcnB* mutant (*t*_1/2_ = 2.8 min for a Δ*pcnB* strain versus *t*_1/2_ = 13.6 min for the Δ*pcnB hfqY25D* strain) but further led to a significant increase in the stability of this sRNA compared to the wild-type strain (*t*_1/2_ = 7.1 min). RyhB stabilities were comparable between an *hfqY25D* single and a Δ*pcnB hfqY25D* double mutant strain (Fig. 4F). In contrast, introduction of the *hfqQ8A* mutation into the Δ*pcnB* strain did not suppress the defect in RyhB stability, and RyhB was rapidly degraded in either an *hfqQ8A* mutant (*t*_1/2_ = 1.97 min) or a Δ*pcnB hfqQ8A* double mutant (*t*_1/2_ = 2.1 min) as compared to a wild-type strain (*t*_1/2_ = 7.1 min) (Table 1; Fig. 4F). This result was consistent with previous studies showing that an alanine substitution in the Hfq proximal face residue Q8 disrupted Hfq binding to RyhB leading to the rapid degradation of this sRNA (Zhang et al. 2013; Schu et al. 2015). Finally, introduction of the *hfqR17A* mutation into a wild-type or a Δ*pcnB* strain background did not significantly decrease RyhB stability relative to that observed in the wild-type strain (Table 1; Supplemental Fig. S5). These data were in agreement with previous studies showing that mutations in the rim-binding residues were not sufficient to lead to a defect in RyhB function (Schu et al. 2015). Overall our data indicated that residue Y25 located on the distal face of Hfq and known to be important for binding targets of RyhB was essential for the decay of RyhB in the absence of PAP I.

### Poly(A) polymerase protects RyhB from target pairing mediated decay

**FIGURE 5.**
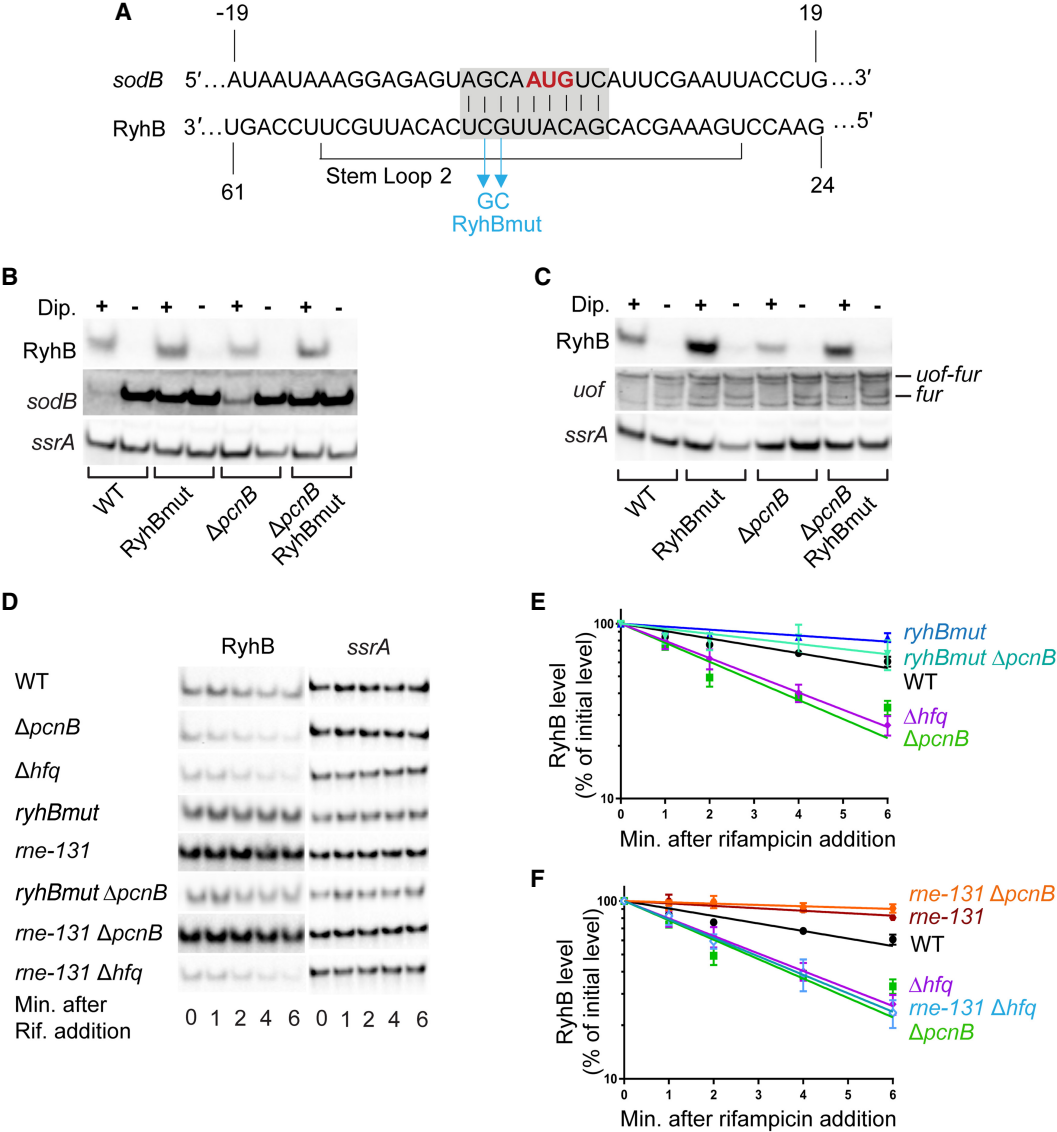
The instability of RyhB in the absence of poly(A) polymerase is due to pairing with target RNAs. (*A*) Schematic showing complementarity (highlighted in gray) between RyhB and its target mRNA *sodB*, and the specific mutations (in cyan) introduced in RyhB to create a RyhB variant unable to pair with target mRNAs (RyhBmut). The start codon of *sodB* is highlighted in red. (*B*,*C*) Northern blot analysis to determine the transcript steady-state levels of RyhB targets *sodB* and *uof* in a wild-type strain (WT [*fur*^+^]; NRD1138) and its derived isogenic mutants (Δ*pcnB*, NRD1198; *ryhBmut*, LM11; *ryhBmut* Δ*pcnB*) under RyhB inducing and noninducing conditions. Overnight cultures of these strains grown in LB were diluted 200-fold in fresh LB media and grown to log phase and treated with dipyridyl (+) to chelate iron or mock-treated (−). After 15 min of treatment, RNA was extracted from each culture and prepared for northern blot analysis to determine corresponding levels of *sodB*, *uof*, RyhB, and *ssrA* (loading control). *uof* transcription is driven from two upstream promoters P_*uof*_ and P_*fur*_ to generate the *uof-fur* and *fur* mRNAs, respectively, but RyhB specifically interacts with the *fur* mRNA (*C*). (*D*–*F*) Determination of RyhB intrinsic stability in a wild-type (WT [*fur*^+^]; NRD1138) and its derived isogenic mutants (Δ*pcnB*, NRD1198; Δ*hfq*, DS021; *ryhBmut*, LM11; *ryhBmut* Δ*pcnB*, LM13; *rne-131*, DS102; *rne-131* Δ*pcnB*, DS106; *rne-131* Δ*hfq*, DS130). Strains were subjected to RNA stability time-course experiments as described in the legend of Figure 4. Representative northern blots are shown in *D*. (*E*,*F*) RyhB decay curves were generated as described in Figure 4 and corresponding half-life measurements are listed in Table 1. Points and error bars in the curves represent the means and the standard errors (SEM) of at least three independent experiments.

RyhB was previously shown to be degraded along with its target RNAs upon sRNA–mRNA pairing (Massé et al. 2003; Lalaouna et al. 2015b). Furthermore, the Y25D substitution in Hfq has been shown to reduce the ability of RyhB to regulate target mRNAs by disrupting Hfq-target RNA interactions (Schu et al. 2015). Thus, based on our results demonstrating the ability of an Hfq^Y25D^ variant to suppress the defect in RyhB stability observed in a Δ*pcnB* strain, we hypothesized that RyhB instability in a Δ*pcnB* strain was a consequence of pairing with target RNAs. To explore this possibility, we constructed a RyhB pairing mutant (*ryhB*mut) in which bases G and C at positions 44 and 45 were inverted (Fig. 5A). These mutations were previously shown to abolish the ability of RyhB to repress the translation of the open reading frame upstream of *fur* (*uof*) and *fur* (Vecerek et al. 2007). Moreover, these nucleotides in RyhB are located in its seed sequence involved in pairing with other targets including *sdhCDAB* and *sodB* mRNAs (Fig. 5A; Massé and Gottesman 2002; Desnoyers and Massé 2012; Peterman et al. 2014; Waters et al. 2017). We assessed whether introducing these mutations into *ryhB* could suppress the defect in RyhB stability observed in a Δ*pcnB* strain by monitoring RyhB or RyhBmut steady-state levels in exponential phase cultures of Δ*pcnB* and *pcnB*^+^ strains by northern blot analysis. Interestingly, introduction of the G44C C45G mutations into *ryhB* in a Δ*pcnB* mutant background suppressed the defect in RyhB levels. Furthermore, RyhB levels increased by approximately 1.5- and twofold in a Δ*pcnB ryhBmut* double mutant and a *ryhBmut* single mutant, respectively, compared to the wild-type strain (Fig. 5B,C). Next, we tested whether the increased levels of RyhB in the Δ*pcnB ryhBmut* strain compared to the Δ*pcnB ryhB*^+^ strain were due to increased stability of the sRNA by monitoring RyhB turnover in exponential phase cultures of these strains after transcription inhibition. As shown in Figure 5D,E and Table 1, the RyhB stability defect observed in a Δ*pcnB* mutant was completely suppressed by introduction of these mutations in the pairing region of RyhB.

In parallel, we tested whether RyhBmut was defective in regulating the *sodB* and *fur* mRNAs. Northern blot analysis showed that in the wild-type strain *sodB* mRNA levels were much lower under RyhB inducing conditions (+dipyridyl) than under noninducing conditions (−dipyridyl) in line with its role in negatively regulating the *sodB* mRNA (Fig. 5B). Consistent with our previous observations, introduction of a *pcnB* deletion in the wild-type background led to a subsequent defect in RyhB-dependent *sodB* regulation as indicated by an increased accumulation of *sodB* transcript under RyhB inducing conditions in a Δ*pcnB* mutant relative to the wild-type strain (Fig. 5B; Supplemental Fig. S6). In contrast, *sodB* mRNA levels were comparable in a *ryhBmut* mutant and a *ryhBmut* Δ*pcnB* double mutant strains under both inducing and noninducing conditions consistent with G44 and C45 of RyhB being critical for sRNA–mRNA pairing. Additionally, we found that the *fur* mRNA, transcribed from the P_*fur*_ promoter located within the *uof* ORF was down-regulated upon RyhB induction in both the wild-type and Δ*pcnB* strains whereas the level of this transcript was unaffected by RyhBmut expression in *ryhBmut* and *ryhBmut* Δ*pcnB* mutant strains (Fig. 5C). Interestingly, RyhB was not able to facilitate the decay of the *uof-fur* mRNA transcribed from the P_*uof*_ promoter located upstream of *uof* ORF, even though RyhB negatively regulated *fur* mRNA levels by interacting with the same site. This finding further suggested that the context of RyhB pairing was important for its interaction with certain target transcripts. Taken together our results confirmed that the G44C C45G mutations in RyhBmut block pairing with at least some target mRNAs, indicating that the rapid turnover of RyhB in the absence of PAP I was dependent upon pairing with target RNAs.

### The C-terminal domain of RNase E is required for RyhB degradation in the absence of poly(A) polymerase

The C-terminal domain (CTD) of RNase E plays an important role in coupled degradation of RyhB and its target mRNAs following Hfq-mediated pairing, and previous studies have demonstrated that this pairing mediated decay can be suppressed by introduction of an internal deletion in *rne* (*rne-131*) (Massé et al. 2003; Prévost et al. 2011; Desnoyers and Massé 2012). This mutation (*rne-131*) leads to production of an RNase E lacking the CTD. The CTD contains two RNA binding regions, ARRBD (or AR1) and AR2, but also interacts with other proteins including enolase, the RNA helicase RhlB, and PNPase to form the RNA degradosome, the central RNA degradation machine (Vanzo et al. 1998; Callaghan et al. 2004; Morita et al. 2005). To further test this hypothesis that absence of PAP I resulted in accelerated decay of RyhB due to increased target pairing, we assessed the ability of an *rne-131* mutant to suppress the defect in RyhB stability in the Δ*pcnB* strain. Introduction of the *rne-131* mutation suppressed the defect in RyhB stability caused by the *pcnB* deletion in both the *fur*^+^ (Table 1; Fig. 5D,F) and Δ*fur* background (Table 1; Fig. 1C,D) leading to a significant increase in RyhB stability in both strains. Additionally, RyhB steady-state levels were up-regulated by 1.9- and 1.5-fold in Δ*fur rne-131* and Δ*fur* Δ*pcnB rne-131* mutants, respectively relative to the Δ*fur* strain (Fig. 1A,B). Interestingly, the *rne-131* mutation failed to suppress the RyhB stability defect in a Δ*hfq* (Table 1; Fig. 5D,F) or a Δ*fur* Δ*hfq* strain background (Table 1; Fig. 1C,D), indicating that the CTD of RNase E was not critical for the degradation of sRNAs not associated with Hfq.

Since introduction of the *rne-131* mutation into the Δ*fur* Δ*pcnB* strain suppressed the defect in RyhB stability, we examined whether this mutation also suppressed the defect in regulation of *sodB* and *sdhCDAB* mRNAs by RyhB. As shown in Figure 3B, we found that introduction of the *rne-131* allele had a modest ability to suppress the defect in RyhB-mediated regulation of *sodB* in the Δ*pcnB* strain (compare 2.2-fold repression in the Δ*fur* Δ*pcnB* strain to 3.9-fold in the Δ*fur* Δ*pcnB rne-131* strain). The ability of the *rne-131* mutation to suppress the defect in RyhB-mediated regulation of the *sdhCDAB* transcript was demonstrated by a 15.8-fold decrease in *sdhC* in the Δ*fur* Δ*pcnB rne-131* strain relative to the Δ*fur* Δ*pcnB rne-131* Δ*ryhB* strain. In contrast, *sdhC* levels were 1.7-fold lower in the Δ*fur* Δ*pcnB* strain relative to the Δ*fur* Δ*pcnB* Δ*ryhB* strain (Fig. 3C).

Finally, we examined whether the defect in the ability of RyhB to regulate *sdhCDAB* mRNA in the Δ*fur* Δ*pcnB* strain relative to the Δ*fur* parental strain had any impact on its ability to grow on succinate as a sole carbon source. As mentioned above, it has been demonstrated that a Δ*fur* mutant is unable to grow on succinate minimal medium due to constitutive RyhB repression of the *sdhCDAB* transcript. As expected, deletion of *hfq* in a Δ*fur* background allowed growth on succinate minimal medium with a yield similar to that observed for a wild-type (*fur*^+^) strain at the end of 24 h and 48 h of growth (Supplemental Fig. S7). Although the growth yields between the Δ*fur* Δ*pcnB* and the Δ*fur* Δ*hfq* strains differed significantly at the end of 24 h, the *pcnB* deletion strain nonetheless reached growth yields comparable to that of an *hfq* deletion strain after 48 h (Supplemental Fig. S7). Consistently, the *rne-131* mutation suppressed the succinate growth phenotype observed for a Δ*fur* Δ*pcnB* strain as indicated by a failure of the Δ*fur* Δ*pcnB rne-131* triple mutant to grow on succinate minimal medium (Supplemental Fig. S7).

### Increased levels of 3′ETS^LeuZ^ drive RyhB decay in the absence of poly(A) polymerase

**FIGURE 6.**
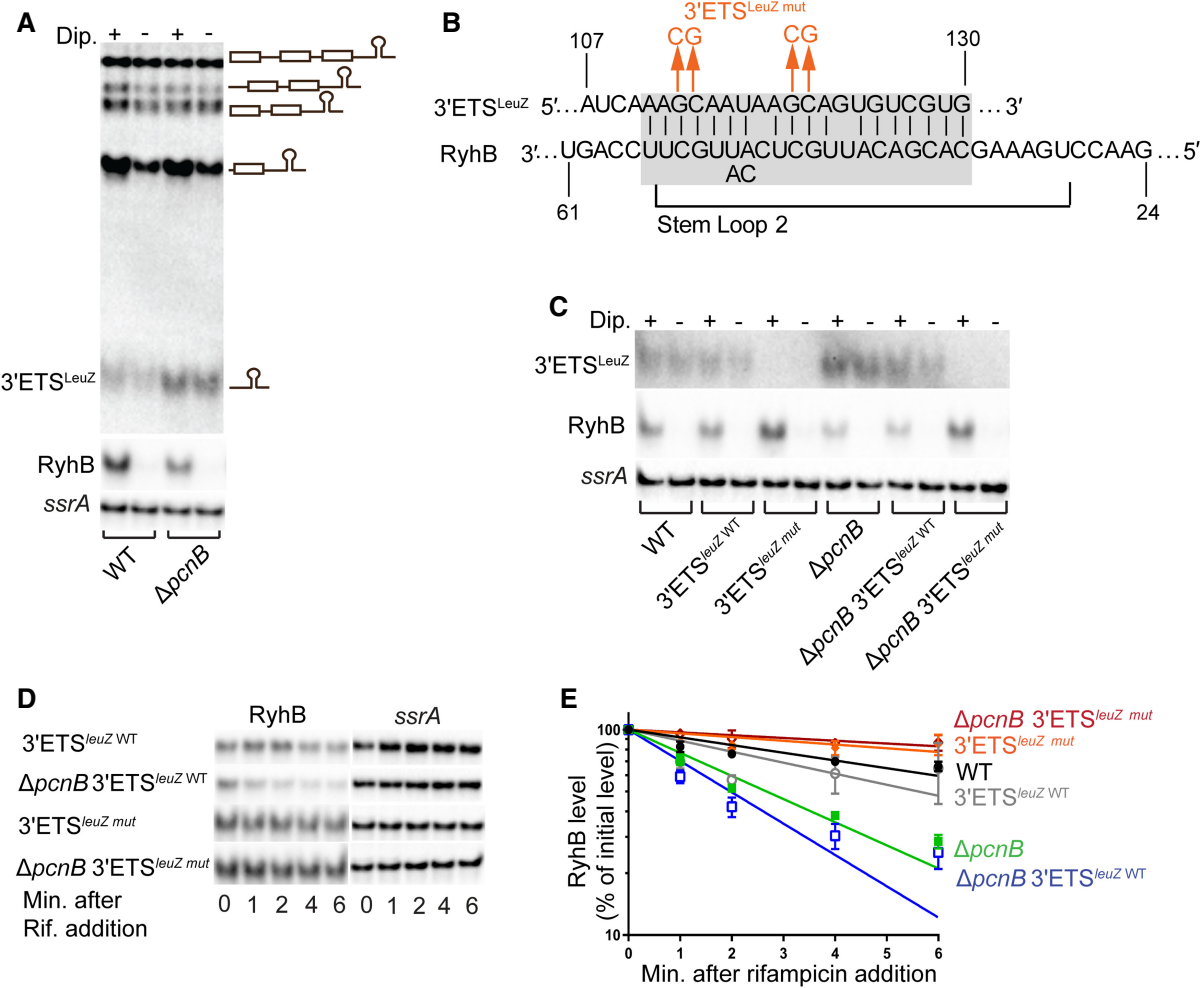
3′ETS^LeuZ^ drives RyhB decay in the absence of poly(A) polymerase. (*A*) Northern blot analysis to determine the steady-state levels of 3′ETS^LeuZ^ and RyhB in a wild-type (WT [*fur*^+^]; NRD1138) and the derived Δ*pcnB* strain (NRD1198) under RyhB inducing and noninducing conditions. Experiments were performed as described in the legend of Figure 5B,C. The primary *glyW*-*cystT*-*leuZ* transcript and the derived processing products that were detected in the northern blot are shown to the *right* of blot image. (*B*) Schematic showing complementarity (highlighted in gray) between RyhB and its target 3′ETS^LeuZ^, and the specific mutations (in orange) introduced in 3′ETS^LeuZ^ to create a LeuZ variant (3′ETS^LeuZ mut^) unable to pair with RyhB sRNA. (*C*) Northern blot analysis to determine steady-state levels of 3′ETS^LeuZ^ in wild-type and derived isogenic *pcnB* and 3′ETS^*leuZ*^ mutant strains under RyhB inducing and noninducing conditions, as described above. Northern blots showing transcript steady-state levels of 3′ETS^LeuZ^ and RyhB in a WT (*fur*^+^; NRD1138) and derived isogenic mutants 3′ETS^*leuZ*^ ^WT^ (*fur*^+^ *leuZ*-*kan*; NRD1579), 3′ETS^*leuZ mut*^ (*fur*^+^ 3′ETS^*leuZ mut*^-*kan*; NRD1581), Δ*pcnB* (*fur*^+^ Δ*pcnB*; NRD1198), Δ*pcnB* 3′ETS^*leuZ*^ ^WT^ (*fur*^+^ Δ*pcnB leuZ*-*kan*; NRD1585), Δ*pcnB* 3′ETS^*leuZ mut*^ (*fur*^+^ Δ*pcnB* 3′ETS^*leuZ mut*^-*kan*; NRD1587). *ssrA* was used as the loading control. Experiment was performed in triplicate and representative blots are shown. LeuZ term probe (Supplemental Table S2) was used to determine LeuZ and 3′ETS^LeuZ^ levels. (*D*,*E*) Determination of RyhB intrinsic stability in a strain encoding the wild-type 3′ETS^LeuZ^ (3′ETS^*leuZ*^ ^WT^; NRD1579) or mutant 3′ETS^LeuZ^ (3′ETS^*leuZ mut*^; NRD1585) and derived Δ*pcnB* strains, NRD1581 and NRD1587, respectively. RNA stability time-course experiments were performed as described in the legend of Figure 4. Representative northern blots are shown in *C*. (*D*) RyhB decay curves were generated as described in Figure 4 and corresponding half-life measurements are listed in Table 1. RyhB decay curves of WT (NRD1138) and its derived isogenic Δ*pcnB* strain (NRD1198) are included in *D* as a reference. Points and error bars in the curves represent the means and the standard errors (SEM) of at least three independent experiments. Probes used are listed in Supplemental Table S2.

Based on our findings, we hypothesized that PAP I facilitates the decay of a certain pool of RyhB target RNAs, which otherwise accumulate in the absence of PAP I, pair with RyhB, and drive its degradation via an RNase E-dependent decay pathway. Recent RNA-seq studies (Maes et al. 2017) identified a potential list of RNAs that serve as substrates for polyadenylation in *E.* coli. One of RyhB targets, the LeuZ precursor tRNA encoded by *leuZ*, was shown to be up-regulated in a Δ*pcnB* mutant (Maes et al. 2017) and to be polyadenylated downstream from the Rho-independent transcription terminator present at the end of its 3′ external transcribed spacer (3′ETS^LeuZ^) (Li and Deutscher 2002; Ow and Kushner 2002). Furthermore, recent studies also demonstrated that overexpression of 3′ETS^LeuZ^ results in reduced levels of RyhB via pairing mediated decay (Lalaouna et al. 2015a,b). Based on these data, we first assessed the steady-state levels of 3′ETS^LeuZ^ in a Δ*pcnB* mutant strain under RyhB inducing (+dipyridyl) and noninducing (−dipyridyl) conditions. Northern blot analysis showed that in the *pcnB* deletion strain, steady-state levels of 3′ETS^LeuZ^ were consistently higher relative to a wild-type strain in both the presence and absence of RyhB induction (Fig. 6A). Next, we investigated whether the increased levels of 3′ETS^LeuZ^ in a *pcnB* mutant strain were driving RyhB decay. To test this, we introduced into *pcnB*^+^ and Δ*pcnB* strains a mutant 3′ETS^LeuZ^ (3′ETS^*leuZ mut*^) in which 4 nt shown to be critical for pairing with RyhB (Lalaouna et al. 2015b) were replaced with the complementary base (Fig. 6B). These nucleotide changes in the 3′ETS^LeuZ^ unexpectedly resulted in a decrease in expression to levels undetectable by northern blots (Fig. 6C; Supplemental Fig. S8); however, this result did not preclude us from assessing the impact of the 3′ETS^LeuZ^ on RyhB expression in the *pcnB*^+^ and Δ*pcnB* strains. To test this, we then assayed for RyhB stability after transcription inhibition in the *pcnB*^+^ and Δ*pcnB* strains that express the wild-type or mutated 3′ETS^LeuZ^. As shown in Figure 6D,E and Table 1, the RyhB stability defect observed in a Δ*pcnB* mutant was completely suppressed by introduction of these mutations in the pairing region of 3′ETS^LeuZ^. Consistently, the introduction of the 3′ETS^*leuZ mut*^ also restored the defect in RyhB steady-state levels of a Δ*pcnB* mutant to wild-type levels (Fig. 6C; Supplemental Fig. S8). Taken together our data demonstrated that the increased decay of RyhB in the absence of PAP I was due to pairing with 3′ETS^LeuZ^.

## DISCUSSION

More than two decades ago, a role for PAP I in mediating the decay of anti-sense sRNAs was discovered (He et al. 1993; Xu et al. 1993). A contemporaneous study demonstrated that PAP I also drives mRNA decay by providing a signal for other ribonucleases (O'Hara et al. 1995). Subsequent work demonstrated a role for PAP I in mediating the decay of anti-sense sRNAs and sRNAs not associated with Hfq (Dam Mikkelsen and Gerdes 1997; Söderbom et al. 1997; Söderbom and Wagner 1998; Szalewska-Palasz et al. 1998; Viegas et al. 2007; Maes et al. 2017). Here, we provide for the first time evidence that PAP I increases the stability of two Hfq-binding sRNAs, RyhB and MicA. Absence of PAP I led to a decrease in RyhB stability (Figs. 1D, 4F) resulting in a defect in RyhB-mediated gene regulation (Fig. 3). These findings indicated a previously unknown role of PAP I in regulating sRNA function and thus raised interesting questions about the mechanism by which this protein can contribute to sRNA stability.

### Mechanism of poly(A) polymerase mediated stabilization of sRNAs

How does PAP I stabilize Hfq-dependent sRNAs such as RyhB? One possibility was that PAP I-dependent stabilization of RyhB resulted from direct polyadenylation of the sRNA leading to an increase in sRNA binding to Hfq, thereby protecting it against RNase E cleavage. This mechanism would be analogous to the mechanism that has been described for PAP I in assisting mRNAs in binding Hfq, i.e., Rho-independent terminators at the 3′ ends of mRNAs are preferentially polyadenylated by the Hfq-PAP I complex, which subsequently results in formation of a stable complex between Hfq and mRNAs (Mohanty and Kushner 2008; Mohanty et al. 2004; Régnier and Hajnsdorf 2013). However, our data does not support this hypothesis. Firstly, we failed to detect poly(A) tails at the ends of full-length RyhB sRNA using 3′ RACE (Supplemental Fig. S9). This result is consistent with the findings of a recent study examining the global landscape of polyadenylated RNAs in *E. coli*, in which no Hfq-dependent sRNAs were among the polyadenylated transcripts identified (Maes et al. 2017). In fact, the sRNAs that were found to be polyadenylated in this study were either anti-sense sRNAs (i.e., sRNAs that regulate mRNAs transcribed from the opposing DNA strand) or sRNAs that bind the RNA chaperone ProQ. Secondly, the relative amounts of RyhB that coimmunoprecipitated with Hfq between a Δ*pcnB* mutant and a wild-type strain under iron starvation were not significantly different (Fig. 4C,D). Altogether, these findings indicated that PAP I mediated stabilization of RyhB was indirect.

Our finding that the RyhB stability defect in a Δ*pcnB* mutant was suppressed by introduction of a Y25D substitution in the distal face of Hfq (Fig. 4E,F) which is known to be important for facilitating sRNA–mRNA target interactions (Zhang et al. 2013; Schu et al. 2015) instead pointed toward a model where PAP I is stabilizing RyhB by protecting it from pairing-mediated decay. Further support for this model came from our results showing that RyhB stability in the Δ*pcnB* mutant increased to wild-type levels (Table 1; Fig. 5D,E) by introduction of a mutation in *ryhB* (*ryhBmut*) that blocked pairing with its target RNAs (Fig. 5A–C).

**FIGURE 7.**
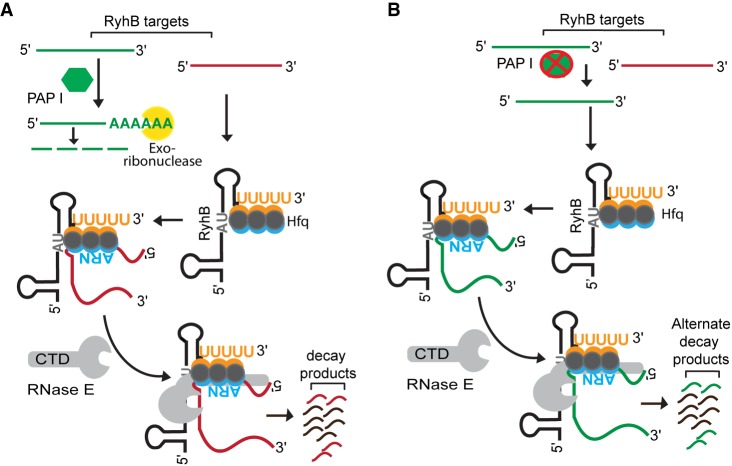
Model for poly(A) polymerase-mediated regulation of RyhB stability. (*A*) Poly(A) polymerase (PAP I) stabilizes RyhB by facilitating the degradation of 3′ETS^LeuZ^ (green-colored RNA) through the addition of poly(A) tails, which provide a toe-hold for exoribonucleases. As a result, RyhB is abundant and able to bind and efficiently regulate other mRNA targets (collectively represented by the red-colored RNA), which are subsequently degraded by RNase E along with RyhB. (*B*) In a strain lacking poly(A) polymerase, 3′ETS^LeuZ^ (green-colored RNA) accumulates and binds to RyhB leading to RNase E-mediated decay of this sRNA. RyhB levels are thereby reduced, and certain mRNA targets (red-colored RNA) accumulate that would otherwise be negatively regulated by this sRNA.

What was the RNA target that was accumulating in the absence of PAP I and driving the decay of RyhB? Over a hundred distinct RNA targets have been shown to be regulated by RyhB (Massé et al. 2005; Lalaouna et al. 2015b; Wang et al. 2015; Melamed et al. 2016); therefore, there were many possible candidates that could have been driving the decay and depletion of RyhB in the Δ*pcnB* strain background. Interestingly, recent work by Maes et al. (2017) identified 14 distinct RNA targets of RyhB that were subjected to polyadenylation in *E. coli.* Furthermore, a subset of these RyhB targets such as *leuZ*, *cspB*, *sdhC*, *sdhD*, *sdhA*, *sdhB*, *fur*, and *uof* were also reported to be up-regulated in the absence of PAP I (Mohanty and Kushner 2006; Maes et al. 2017). Of note here is the LeuZ precursor tRNA encoded by *leuZ*, which was one of the RyhB targets that was highly up-regulated in a Δ*pcnB* mutant (Maes et al. 2017). Past studies also pointed toward a critical role of PAP I in promoting the degradation of the 3′ETS^LeuZ^ by exoribonucleases following its excision from the LeuZ precursor tRNA by RNase E (Li and Deutscher 2002; Ow and Kushner 2002). More importantly, it was recently shown that 3′ETS^LeuZ^ can pair with RyhB sRNA and down-regulate RyhB transcript levels, which in turn leads to decreased regulation of RyhB targets including the *sodB* and *sdhCDAB* mRNAs (Lalaouna et al. 2015a,b). Based on these findings, we hypothesized that PAP I promotes the exoribonucleolytic decay of the 3′ETS^LeuZ^ through polyadenylation of this transcript (Fig. 7A). Furthermore, we postulated that in the absence of PAP I, 3′ETS^LeuZ^ accumulates and drives RNase E-mediated decay of RyhB as a result of base-pairing with this sRNA leading to loss of regulation of its other target mRNAs including *sodB* and *sdhCDAB* (Fig. 7B). In support of this hypothesis, we found that introduction of a mutation in 3′ETS^*leuZ*^ (3′ETS^*leuZ mut*^) that was previously shown to block pairing of 3′ETS^LeuZ^ with RyhB sRNA (Lalaouna et al. 2015b) suppressed the RyhB stability defect of a Δ*pcnB* mutant (Table 1; Fig. 6D,E) and led to a significant increase in RyhB steady-state levels (Fig. 6C; Supplemental Fig. S8). Decreased RyhB stability in the Δ*pcnB* mutant subsequently resulted in an impaired regulation of the *sodB* and *sdhCDAB* mRNAs (Figs. 3, 5B; Supplemental Fig. S2, S3, S6, S7). This reduced regulation of *sdhCDAB* by RyhB in the absence of PAP I has a measurable impact on *E. coli* physiology resulting in the ability of a Δ*pcnB* Δ*fur* strain to grow on succinate as the sole carbon source (Supplemental Fig. S7).

Interestingly, several examples have now been described of target RNAs that drive the decay of sRNAs resulting in reduced regulation of other mRNAs. The first reported example in this category in *E. coli* and *Salmonella enterica* was described by Plumbridge et al. (2014) who demonstrated that pairing of an Hfq-dependent sRNA (ChiX) with one target mRNA (*chbBC*) drove the decay of that sRNA. This depletion of ChiX resulted in impaired regulation of another one of its targets, the *chiP* mRNA (Figueroa-Bossi et al. 2009). Subsequently, Miyakoshi et al. (2015) demonstrated that the SroC sRNA generated in the decay of the *gltIJKL* transcript in *E. coli* and *Salmonella enterica* functioned to titrate GcvB levels (Miyakoshi et al. 2015).

### Mechanisms by which poly(A) polymerase and Hfq block RNase E-mediated decay of sRNAs are distinct

The endoribonuclease RNase E is a key protein involved in degrading both Hfq-dependent sRNAs and cleaving target mRNAs upon sRNA–mRNA pairing (De Lay et al. 2013). The N-terminal domain of RNase E contains the catalytic site, but can also bind RNA through its S1 domain and the 5′ sensor domain as well as its active site residues (Garrey et al. 2009). The C-terminal domain (CTD) of RNase E contains two RNA binding sites, the ARRBD and AR2 regions, in addition to binding sites for other proteins including enolase, the RNA helicase RhlB, and the 3′ → 5′ exoribonuclease PNPase (Mackie 2013). The results of two previous studies suggested that Hfq interacts directly with the CTD of RNase E (Morita et al. 2005; Ikeda et al. 2011), but detailed biochemical studies indicate that Hfq interacts with the CTD via RNA (Worrall et al. 2008). The CTD was also previously shown to be important for coupled degradation of RyhB with its target mRNAs (Massé et al. 2003; Desnoyers and Massé 2012). It is notable that the defect in RyhB stability that was observed in a Δ*pcnB* mutant was suppressed by introduction of an *rne-131* allele encoding an RNase E lacking the CTD (Figs. 1C,D, 5D,F), whereas introduction of an *rne-131* mutation failed to suppress the RyhB stability defect of an Δ*hfq* mutant (Figs. 1C,D, 5D,F). Since RyhB decay in the Δ*pcnB* strain was due to pairing with the 3′ETS^LeuZ^, these results demonstrated a requirement for the CTD of RNase E for degradation of RyhB upon pairing with the 3′ETS^LeuZ^, which may indicate that one or both of the RNA binding regions, ARRBD and AR2, mediate the recruitment of this sRNA to RNase E after pairing with target RNAs. However, an alternative interpretation of our results is that the RNA degradosome was required for the degradation of RyhB after sRNA–mRNA pairing as the CTD is important for the assembly of the RNA degradosome, which includes enolase, the RNA helicase RhlB, and PNPase. In contrast, the inconsequential role of the CTD in decay of RyhB in the Δ*hfq* strain may signify that the N-terminal catalytic domain of RNase E is sufficient to cleave RyhB when it is not associated with Hfq or RNA targets.

### Impact of poly(A) polymerase on a subset of Hfq-dependent sRNAs

As mentioned above, sRNAs can be classified into two distinct classes based on their interactions with Hfq (Zhang et al. 2013; Schu et al. 2015). Class I sRNAs interact with proximal face and rim of Hfq, and their mRNA targets bind the distal face of Hfq (Fig. 4E). In contrast, Class II sRNAs interact with the proximal and distal faces of Hfq, and their target mRNAs bind along the rim. The fact that deletion of *pcnB* led to a defect in the stability of MicA in addition to RyhB (two Class I sRNAs) but did not significantly impact the turnover of GcvB or Class II sRNAs such as ChiX, CyaR, and MgrR (Fig. 2A–E; Supplemental Fig. S1) is interesting. Whether this difference in the ability of PAP I to impact the stability of MicA and RyhB, but not ChiX, CyaR, and MgrR was due to the fact that these sRNAs interact with Hfq differently or was a coincidence will need to be resolved in future studies. Regardless, the ability of PAP I to impact a specific subset of sRNAs by a mechanism that is distinct from Hfq suggests an additional layer of complexity in sRNA-mediated gene regulation.

## MATERIALS AND METHODS

### Bacterial strains and growth conditions

All strains and plasmids used in this study are derivatives of *E. coli* K-12 strain MG1655 (*rph-1*) and are listed in Supplemental Table S1. Primers used for strain construction are listed in Supplemental Table S2. Strain construction is described in Supplemental Materials and Methods.

All strains were grown in liquid medium or agar plates containing either Lennox broth (LB), M9 minimal medium supplemented with 0.001% vitamin B1 and 0.2% glucose or succinate, or MOPS EZ rich defined medium (Teknova) supplemented with 0.4% glycerol instead of glucose. Antibiotics were used in the following final concentrations: ampicillin, 100 µg/mL; kanamycin, 25 µg/mL; chloramphenicol, 25 µg/mL or 10 µg/mL (for any mutant(s) containing *hfq* deletion), tetracycline, 12.5 µg/mL, zeocin, 25 µg/mL, and rifampicin, 250 µg/mL. 2, 2′ dipyridyl was added to liquid medium at a final concentration of 250 µM. All liquid cultures and bacteria on solid medium were grown aerobically at 37°C. Overnight cultures were diluted 1:200 fold in appropriate medium and grown until desired densities were reached. Growth was determined by measuring the optical densities of liquid cultures at 600 nm (OD_600_). Cultures were considered to be in exponential phase when they reached OD_600_ between 0.3 and 0.4 in LB or OD_600_ of ∼1.0 in MOPS EZ rich defined medium.

### RNA extraction

Total RNA was extracted from exponential phase cultures growing either in LB or in MOPS EZ rich defined medium using hot phenol lysis method described previously (Massé et al. 2003). Briefly 700 µL of samples were removed from growing cultures and added to a mixture containing 800 µL of acid phenol–chloroform-isoamyl alcohol (pH of 4.3; Fisher Scientific) and 100 µL of lysis buffer (320 mM sodium acetate [pH 4.6], 8% SDS, 16 mM EDTA) equilibrated to 65°C. Samples were mixed at 65°C for 5 min and centrifuged for 30 min at 4°C to separate phases. The upper aqueous phase was extracted a second time with equal volume of neutral phenol–chloroform-isoamyl alcohol (pH of 6.7; Fisher Scientific). RNA was alcohol-precipitated and resuspended in DEPC-treated water. RNA concentration was measured using Nano Drop 2000 (Thermo Fisher Scientific).

### RNA stability assay

To determine RNA stabilities for sRNAs in different mutants constructed in the *fur*^+^ background (Supplemental Table S1) all cultures were grown to exponential phase. RyhB or MicA expression were induced from a *ryhB* promoter by the addition of dipyridyl (iron chelator) while CyaR expression was induced from its native promoter by the addition of cyclic AMP (Adenosine 3′, 5′- cyclic monophosphate sodium salt monohydrate, Sigma) at a 5 mM final concentration. Under exponential growth conditions expression of MgrR, GcvB, and ChiX is constitutive. After 15 min of sRNA expression a culture sample (T_0_) was collected. Following that, rifampicin was added to inhibit all further transcription and additional samples were collected 1, 2, 4, and 6 min after rifampicin addition. To determine RyhB stability in mutants constructed in a *fur* deletion background strains were grown in MOPS EZ rich defined medium to exponential phase and a culture sample was collected (T_0_). Rifampicin was added to all cultures to inhibit transcription and additional samples were collected at 2, 5, 10, 15, and 30 min after rifampicin addition. All samples were subjected to RNA extraction as described above.

### Northern blot analysis

Two micrograms of each RNA sample was loaded on 5% or 10% Criterion TBE-urea precast gels (Bio-Rad) and electrophoresed at 70 V. Next, the RNA samples were transferred to a Zeta-Probe GT membrane (Bio-Rad) using a Trans-Blot SD semidry transfer apparatus (Bio-rad) following manufacturer's guidelines. Transferred RNA was UV crosslinked and hybridized overnight with 100 ng/mL of 5′ biotinylated DNA probe (Supplemental Table S2) in ULTRAhyb (Ambion) hybridization buffer at 42°C. Blots were developed using a BrightStar BioDetect kit protocol (Ambion), imaged with a ChemiDoc MP imager (Bio-Rad) and quantified using Image Lab software version 5.2.1 (Bio-Rad). Signal intensity corresponding to each sRNA or mRNA was normalized to that of either *ssrA* or 5S rRNA, which served as internal loading controls. Decay curves corresponding to RNA stability time course experiments were generated by using GraphPad Prism version 5.0.

### Quantitative RT-PCR (qRT-PCR) analysis

RNA extracted from exponential growth phase cultures as described above was subjected to DNase treatment (DNase Turbo; Ambion) following manufacturer's protocol. Sample mixtures (total reaction volume of 100 µL) were incubated for 1 h at 37°C and reaction was stopped by addition of 100 µL of DEPC-treated water and 200 µL of neutral phenol–chloroform-isoamyl alcohol (Fisher). DNase treated RNA samples were phenol extracted, alcohol precipitated and RNA concentration was measured as described above. Samples were then tested for the presence of any contaminating DNA by PCR using gene specific qRT-PCR primers (Supplemental Table S2) before proceeding to downstream applications. One microgram of DNA-free RNA was reverse transcribed using random hexamers and Superscript III Reverse Transcriptase (RT) (Invitrogen) following manufacturer's protocol. For each sample a no RT (NRT) control reaction was performed. cDNA samples were diluted 1:100-fold and 2 µL of diluted samples (resulting from both RT- and NRT- PCR) were used in a qRT-PCR reaction mixture containing 10 µL of iTaq Universal SYBR Green Supermix (Bio-Rad) and 2 µL each of 4 µM qPCR primers (Supplemental Table S2). A single no template control (NTC) for each qPCR primer pair used in this study was also included. Data were collected using an CFX connect Real Time thermocycler (Bio-Rad) running the SYBR Green with melt curve program modified as per the manufacturer's recommendations. Reactions were performed with two technical duplicates using cDNA samples from at least three independent biological replicates per strain and *ssrA* was used as the internal reference for normalization. The ΔΔC_t_ method was used to calculate fold changes of transcripts corresponding to target genes in different sets of mutants relative to the wild-type parent. Statistical analysis was performed using one-way ANOVA (and nonparametric) in GraphPad Prism version 5.0.

### Protein extraction and western blot analysis

Overnight cultures of wild-type and mutant strains were subcultured into fresh 10 mL LB and grown to exponential phase. Protein extraction was performed as described previously (De Lay and Gottesman 2011). Briefly, 1.0 mL of culture from each strain was subjected to TCA precipitation, washed once with 80% cold acetone solution, air dried, suspended in 2× Laemmli sample buffer (Bio-Rad) containing freshly added 5% (vol/vol) β-mercaptoethanol and heated at 95°C for 10 min. Total protein amount was normalized to corresponding OD_600_ of each specific culture. Approximately 0.08 OD_600_ units of total protein from each strain was separated on a 4% stacking 10% resolving SDS-PAGE gel in 1× Tris-glycine SDS buffer at 120 V. Fractionated protein was then transferred to a 0.45 μm PVDF membrane (Thermo Scientific) at 15 V for 30 min using Trans-Blot SD semidry transfer apparatus (Bio-rad) following manufacturer's guidelines.

Hfq was detected using 1:5000 dilution of preabsorbed anti-Hfq antiserum obtained from Dr. Susan Gottesman (NCI) and goat anti-rabbit IgG secondary antibody. For detection of DnaK (loading control) 1:10,000 dilution of mouse anti-DnaK monoclonal antibody (Abcam) and anti-mouse goat secondary antibody (Santa Cruz Biotechnologies, Inc.) were used following manufacturer's guidelines. All secondary antibodies were conjugated to alkaline phosphatase and were visualized by using Immun-Star AP substrate (Bio-Rad) and ChemiDoc MP imager (Bio-Rad). Signal intensity was quantitated using Image Lab software (Bio-Rad).

### Hfq coimmunoprecipitation

Immunoprecipitation with Hfq was performed as described previously (Bandyra et al. 2016) with few modifications. Briefly, overnight cultures were diluted 200-fold into 30 mL of fresh LB liquid medium and grown to an OD_600_ of ∼0.3. Dipyridyl was added to induce RyhB expression for 15 min and 25 mL aliquots of cells were pelleted, washed, and frozen as previously described (Zhang et al. 2003). Immunoprecipitations were performed as previously described (Zhang et al. 2002) using anti-Hfq antiserum obtained from Dr. Susan Gottesman (NCI). RNA was isolated by phenol extraction and northern blots were performed as described in Materials and Methods.

## SUPPLEMENTAL MATERIAL

Supplemental material is available for this article.

## Supplementary Material

Supplemental Material
