# Supplementary material for: Poly(A) polymerase is required for RyhB sRNA stability and function in Escherichia coli
Source: RNA. 2018 Nov;24(11):1496–511. doi: 10.1261/rna.067181.118 (PMC6191717; doi:10.1261/rna.067181.118)
Supplement: Supplemental Material [file supp_24_11_1496__index.html]

Poly(A) polymerase is required for RyhB sRNA stability and function in Escherichia coli — Poly(A) polymerase is required for RyhB sRNA stability and function in Escherichia coli — Supplemental Material 

# Poly(A) polymerase is required for RyhB sRNA stability and function in *Escherichia coli*

## Supplemental Material

- Supplemental\_Materials.pdf
