# Supplementary material for: Poly(A) polymerase is required for RyhB sRNA stability and function in Escherichia coli
Source: RNA. 2018 Nov;24(11):1496–511. doi: 10.1261/rna.067181.118 (PMC6191717; doi:10.1261/rna.067181.118)
Supplement: Supplemental Material [file supp_067181.118_Supplemental_Materials.pdf]

SUPPLEMENTAL MATERIALS

Poly(A) polymerase is required for RyhB sRNA stability and function in *Escherichia coli*

Dhriti Sinha[1], Lisa M. Matz[1], Todd A. Cameron[1] and Nicholas R. De Lay[1, 2*]

[1]Department of Microbiology and Molecular Genetics, McGovern Medical School, University of Texas Health Science Center, Houston, TX 77030, USA

[2]MD Anderson Cancer Center UTHealth Graduate School of Biomedical Sciences, University of Texas Health Science Center, Houston, TX 77030, USA

[*] Correspondence should be sent to Nicholas R. De Lay. Tel: +1 (713) 500-6293; Email: nicholas.r.delay@uth.tmc.edu

Running title: Poly(A) polymerase promotes sRNA stability

Key words: Poly(A) polymerase, *pcnB*, Hfq, small RNAs, RNase E

TABLE OF CONTENTS

TABLE S1. Strains and plasmid used in this study.

TABLE S2. Primers and probes used in this study.

FIGURE S1. Representative Northern blots corresponding to sRNA stability curves shown in Figure 2.

FIGURE S2. Northern Blot analysis of *sodB* mRNA turnover in *pcnB* mutant.

FIGURE S3. Analysis of the effects of deletions in *pcnB*, *hfq* and *rne* on *sdhD* transcript steady-state levels in an *E. coli* Δ*fur* strain**.**

FIGURE S4. Determination of the transcript steady-state levels of MicA targets *ompA* and *ompX* in *pcnB* mutant.

FIGURE S5. Northern blot analysis to determine RyhB stability in the wild-type and its derived isogenic *hfq* and *pcnB* mutants.

FIGURE S6. Determination of the relative abundance of *sodB* transcript in wild-type (*fur*$^{+}$; WT) and its derived isogenic *fur* and *pcnB* mutants.

FIGURE S7. The defect in regulation of the *sdhCDAB* mRNA by RyhB in the absence of poly(A) polymerase consequently impacts the ability of *E. coli* to utilize succinate as the sole carbon source.

FIGURE S8. Northern blot analysis to determine transcript steady-state levels of LeuZ, LeuZ$^{3'ETS}$ and RyhB in wild-type and derived isogenic *pcnB* and *leuZ* mutants under RyhB inducing and non-inducing conditions.

FIGURE S9. Analysis of 3′-end polyadenylation state of RyhB.

SUPPLEMENTAL MATERIALS AND METHODS

SUPPLEMENTAL REFERENCES

TABLE S1. Strains and Plasmid

Strains or plasmids	Relevant features	Reference or Source
**Strains**		
MG1655	*rph-1*	(Blattner et al. 1997)
KR10000	MG1655 *rph*$^+$	D. Court, NCI
NM600T	*rph-1* Δ*lacZ* mini tet lambda	N. Majdalani, NCI
EM1238	*rph-1* Δ*lacX74* Δ*ryhB::cat*	(Masse et al. 2003)
EM1377	*rph-1* Δ*lacX74 rne -131 zce-726*::Tn*10*	(Masse et al. 2003)
JW3617	*rph-1* Δ(*araD-araB*)*567* Δ(*rhaD-rhaB*)*568* Δ*lacZ4787* (::*rrnB-3*) *hsdR514* Δ*pyrE*::*kan*	(Baba et al. 2006)
JW4130	*rph-1* Δ(*araD-araB*)*567* Δ(*rhaD-rhaB*)*568* Δ*lacZ4787* (::*rrnB-3*) *hsdR514* Δ*hfq*::*kan*	(Baba et al. 2006)
JW5808	*rph-1* Δ(*araD-araB*)*567* Δ(*rhaD-rhaB*)*568* Δ*lacZ4787* (::*rrnB-3*) *hsdR514* Δ*pcnB*::*kan*	(Baba et al. 2006)
DJS2812	*rph-1* Δ*lacX74 mal*::*lacI*^{q} Δ*araBAD* Δ*hfq*::*cat-sacB* Δ*purA*::*kan*	(Schu et al. 2015)
DJS2817	*rph-1* Δ*lacX74 mal*::*lacI*^{q} Δ*araBAD hfqQ8A*	(Schu et al. 2015)
DJS2820	*rph-1* Δ*lacX74 mal*::*lacI*^{q} Δ*araBAD hfqR17A*	(Schu et al. 2015)
DJS2864	*rph-1* Δ*lacX74 mal*::*lacI*^{q} Δ*araBAD hfqY25D*	(Schu et al. 2015)
TC279	*rph-1* Δ*cyaR* Δ*micA*::*zeo* Δ*ryhB*::*micA*	(Cameron and De Lay 2016)
NM600T	*rph-1* Δ*lacZ* mini-λ tet	N. Majdalani, NCI
NM1200	*rph-1* mini-λ cm (*cat*)	N. Majdalani, NCI
NRD463	*rph-1* Δ*pnp*::*cat*	(De Lay and Gottesman 2011)
NRD473	*rph-1* Δ*lacX74 mal*::*lacI*^{q} Δ*pnp*::*kan*	(De Lay and Gottesman 2011)
NRD576	*rph-1* Δ*lacX74 mal*::*lacI*^{q} Δ*pnp*::*kan rne-131 zce-726*::Tn*10*	NRD473 + P1(EM1377)
NRD698	*rph-1* Δ*lacZ* Δ*pcnB*::*cat*	This study
NRD999	*rph*$^+$ Δ*pnp*::*cat*	KR10000 + P1(NRD463)
NRD1038 λ	*rph-1* Δ*ryhB*::kan-pBAD-*ccdB* mini-λ tet	This study
NRD1138	*rph-1*	(Cameron and De Lay 2016)
NRD1198	*rph-1* Δ*pcnB*::*cat*	NRD1138 + P1 (NRD698)
NRD1243	*rph*$^+$ *pnp*-3XFLAG	(Bandyra et al. 2016)
NRD1362	*rph*$^+$ Δ*fur*::*zeo*	This study
NRD1410	*rph-1 hfqY25D*	DS057 + P1 (DJS2864)
NRD1455	*rph-1* Δ*malQ*::*nptII*	NRD1138 + P1(JW3379)
NRD1530	*rph-1* Δ*asnB*::*tet*	This study
NRD1533	*rph-1* Δ*asnB*::*tet* Δ*fur*::*zeo*	NRD1530 + P1 (NRD1362)

NRD1546	*rph-1 ΔryhB::cat Δfur::zeo*	DS132 + P1(NRD1533)
NRD1547	*rph-1 ΔpcnB::kan ΔryhB::cat Δfur::zeo*	DS135 + P1(NRD1533)
NRD1550	*rph-1 rne -131 zce-726*::Tn*10 ΔryhB::cat Δfur::zeo ΔpcnB::kan*	DS137 + P1(NRD1362)
NRD1551	*rph-1 rne -131 zce-726*::Tn*10 ΔryhB::cat*	DS137 + P1(JW5808)
NRD1576	rph^{+} *leuZ3'ETS-kan*	This study
NRD1578	rph^{+} *leuZ3'ETS* G113C C114G G120C C121G-*kan*	This study
NRD1579	*rph-1 leuZ3'ETS-kan*	NRD1138 + P1 (NRD1576)
NRD1581	*rph-1 leuZ3'ETS* G113C C114G G120C C121G-*kan*	NRD1138 + P1 (NRD1578)
NRD1585	*rph-1 ΔpcnB::cat leuZ3'ETS-kan*	NRD1198 + P1 (NRD1576)
NRD1587	*rph-1 ΔpcnB::cat leuZ3'ETS* G113C C114G G120C C121G-*kan*	NRD1198 + P1 (NRD1578)
DS021	*rph-1 Δhfq::kan*	NRD1138 + P1 (JW4130)
DS024	*rph-1 Δfur::zeo*	NRD1138 + P1 (NRD1362)
DS025	*rph-1 ΔpcnB::cat Δfur::zeo*	NRD1198 + P1 (NRD1362)
DS027	*rph-1 Δhfq::kan Δfur::zeo*	DS021 + P1 (NRD1362)
DS057	*rph-1 Δhfq::cat-sacB ΔpurA::kan*	NRD1138 + P1 (DJS2812)
DS058	*rph-1 hfqQ8A*	DS057 + P1 (DJS2817)
DS060	*rph-1 hfqR17A*	DS057 + P1 (DJS2820)
DS071	*rph-1 ΔpcnB::kan*	NRD1138 + P1 (JW5808)
DS072	*rph-1 hfqQ8A ΔpcnB::cat*	DS058 + P1 (NRD1198)
DS073	*rph-1 hfqR17A ΔpcnB::cat*	DS060 + P1 (NRD1198)
DS069	*rph-1 Δfur::zeo rne-131 zce*-726::Tn10	DS024 + P1 (EM1377)
DS082	*rph-1 Δfur::zeo ΔpcnB::cat rne-131 zce*-726::Tn10	DS025 + X P1 (EM1377)
DS089	rph^{+} *pnp*-3XFLAG *ΔpyrE::kan*	NRD1243 + P1 (JW3617)
DS090	*rph-1 pnp*-3XFLAG	DS089 + P1 (MG1655)
DS092	*rph-1 pnp*-3XFLAG *ΔpcnB::cat*	DS090 + P1 (NRD1198)
DS102	*rph-1 rne -131 zce-726*::Tn*10*	NRD1138 + P1 (EM1377)
DS106	*rph-1 rne -131 zce-726*::Tn*10 ΔpcnB::cat*	NRD1198 + P1 (EM1377)
DS120	*rph-1 ΔcyaR ΔmicA::zeo ΔryhB::micA ΔpcnB::kan*	TC279 + P1 (JW5808)
DS130	*rph-1 Δhfq::kan rne -131 zce-726*::Tn*10*	DS021 + P1 (EM1377)
DS132	*rph-1 ΔryhB::cat*	NRD1138 + P1 (EM1238)
DS135	*rph-1 ΔpcnB::kan ΔryhB::cat*	DS071 + P1 (EM1238)
DS137	*rph-1 rne -131 zce-726*::Tn*10 ΔryhB::cat*	DS102 + P1 (EM1238)
DS153	*rph-1 Δhfq::kan rne -131 zce-726*::Tn*10* Δfur::*zeo*	DS130 + P1 (NRD1362)
DS185	*rph-1 hfqY25D ΔpcnB::cat*	NRD1410 + P1 (NRD1198)
LM06	*rph-1 ryhB* G44C C45G	This study
LM10	*rph-1 ΔmalQ ΔlacX*74 *ΔryhB::cat*	NRD1455 + P1(EM1238)
LM11	*rph-1 ryhB* G44C C45G	LM10 + P1(LM06)
LM13	*rph-1 ryhB* G44C C45G *ΔpcnB::cat*	LM11 + P1(NRD1198)
**Plasmid**		
pKD3	Amp^r, Cm^r; oriR6Kɣ; *cat* cassette flanked by FRT sites	(Datsenko and Wanner 2000)
pKD4	Amp^r, Kan^r; oriR6Kɣ; *kan* cassette	(Datsenko and Wanner 2000)

	flanked by FRT sites	

TABLE S2. Primers and Probes

	**Sequence (5′ - 3′)**
**Probes**	
RyhB	AAGTAATACTGGAAGCAATGTGAGCAATGTCGTGCTTTCAGGTTCT C
RyhB-LM	GTCGTGCTTTCAGGTTCTCCGCGAGGGTCTTCC
MicA	CCAAAATTTCATCTCTGAATTCAGGGATGATGATAACAAATG
GcvB	CCAGAACACGCATTCCGATAAAACTTTTCGTTCCGGCTCAGG
CyaR	TGGTTCCTGGTACAGCTAGCATTTTATGGGTTATG
ChiX	CATTTTTTTATTATTATGCCGTCACTTTAAGCGACGGTG
MgrR	CAGTAAACCGGCGGTGAATGCTTGCATGGATAGAT
SsrA	CGCCACTAACAAACTAGCCTGATTAAGTTTTAACGCTTCA
5S	ATGGGGTCAGGTGGGACCACCGCGCTACTGC
sodB	TGCGATAGTCGATGTAATAAGCGTGTTCCCAGACATCAAC
sodB 5'-UTR	GGTAGTGCAGGTAATTCGAATGACATTGCTACTCTCC
uof	GCTCTTGAGATAATGCGTATCATTATAGAATTGCCACGCC
ompA	CCATTGTTGTTGATGAAACCAGTGTCATGGTACTGGGACCAGC
ompX	TACGGAAGTACCTGCGGTGAAAGCCAGAACTGCGG
3'ETS^{LeuZ}	CCACCCGAAGGTGGTTTCACGACACTGCTTATTGCTTTG
LeuZ term	AAAAAAACCACCCGAAGGTGGTTTCACGACACT
16S	CCTGTTACCGTTCGACTTGC
**qRT-PCR primers**	
q*sdhC*_For	CGATAGCGTCCATTCTCCATC
q*sdhC*_Rev	GACGAAGAAGCTGCCCATAA
q*sdhD*_For	TATCGTCCTGACGCTCTACA
q*sdhD*_Rev	CCAGGCATGGATCAAGATAGAA
q*ssrA*_For	TTTGTTAGTGGCGTGTCCGT
q*ssrA*_Rev	GAACCCGCGTCCGAAAT
**Primers for strain construction**	
fur::zeo For	TCTAATGAAGTGAACCGCTTAGTAACAGGACAGATTCCGCTGTTG ACAATTAATCATCGGC
fur:: zeo Rev	AAAGCCAACCCGCAGGTTGGCTTTTCTCGTTCAGGCTGGCGGGTG GTCTTAATCTGTTTATC
leuZKOchk For	CGGATGTCTGGCTGAAAGG
leuZKOchk Rev	CCAGCACCTATCTTACATGCC
leuZwtKOin For	CCGGGTACCATGGGAAAGATAAGAATAAAATCAAAGCAATAAGCA GTGTCGTGAAACCACCTTCGGGTGGTTTTTTTGTGTAGGCTGGAG CTGCTTC
leuZKOin Rev	GGTGATTAAAAATTAAGGAGGGTGTAACGACAAGTTGCAGCATAT GAATATCCTCCTTAG

leuZdblinvKOin For	CCGGGTACCATGGGAAAGATAAGAATAAAATCAAACGAATAACGA GTGTCGTGAAACCACCTTCGGGTGGTTTTTTTGTGTAGGCTGGAG CTGCTTC
pcnB KO For	TTTTTGTCCTGAATGATGTTTGACACTACCGAGGTGTACTGTGTAG GCTGGAGCTGCTTC
pcnB KO Rev	CCTATGGCAATATACGCCACTGTCATGCGGTACCCTCACGCATAT GAATATCCTCCTTAG
pcnB For	GTGGCTTTCAGGGATATCG
pcnB Rev	CTTTCCAAGCAGAATCATGCG
furKOchk For	GTGGCAATTCTATAATGATACGC
furKOchk Rev	GAGAGCTGTAACTCTCGC
RyhBKOchk For	CAAATGCGAGTCAAATGC
RyhBKOchk Rev	GTGGTTCCTACTGGAGTGC
RyhBmut gBlock®	GTGTTGGACAAGTGCGAATGAGAATGATTATTATTGTCTCGCGATC AGGAAGACCCTCGCGGAGAACCTGAAAGCACGACATTCGTCACAT TGCTTCCAGTATTACTTAGCCAGCCGGGTGCTGGCTTTTTTTTTGA TCTTTCGTTCTCAATTTATCCACGGGAGTGCTTGTGTT
**3′ RACE primers**	
RyhB3'RACE	GCGATCAGGAAGACCCTC
E1 DNA adapter	TTCACTGTTCTTAGCGGCCGCATGCTC
M13 For	GTAAAACGACGGCCAG
M13 Rev	CAGGAAACAGCTATGAC

SUPPLEMENTAL FIGURES

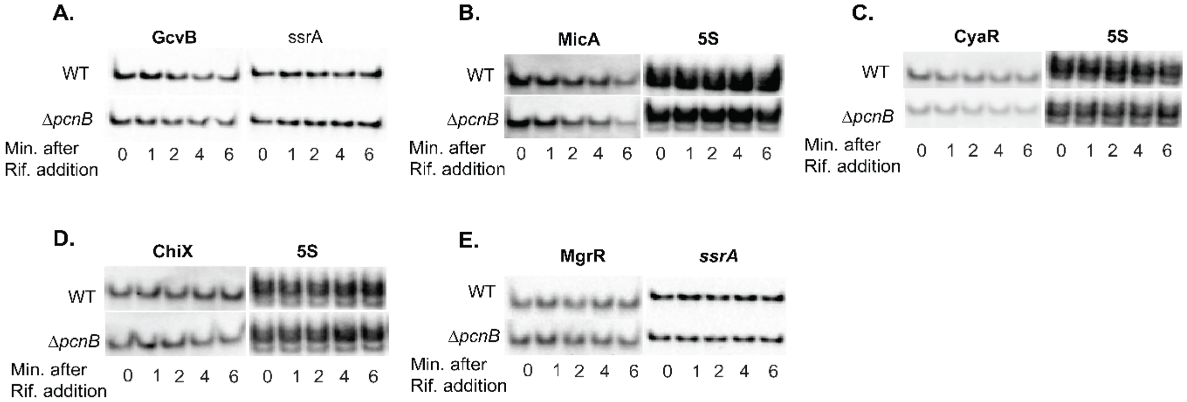

FIGURE S1. Representative northern blots corresponding to sRNA stability curves shown in Figure 2. Northern blot analysis was used to determine the expression of the following sRNAs: GcvB, MicA, CyaR, ChiX, and MgrR, and their corresponding loading controls (SsrA or 5S) at the indicated time points following rifampicin addition in strains TC279 (A and B), NRD1138 (C and D), or the derived Δ*pcnB* mutant strains DS120 and NRD1198, respectively, under exponential growth conditions as described in the legend of Figure 2 and in Materials and Methods.

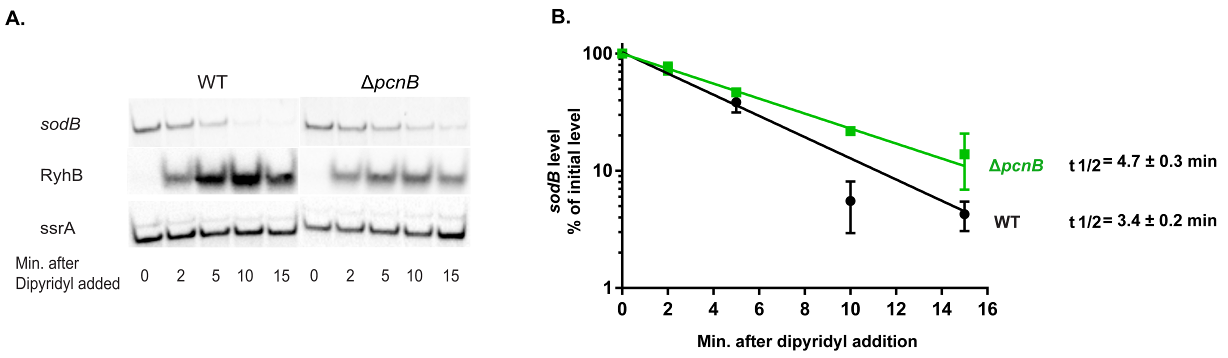

FIGURE S2. Northern Blot analysis of *sodB* mRNA turnover in *pcnB* mutant. RyhB expression was induced in exponentially growing cultures of the wild-type (NRD1138) and Δ*pcnB* (NRD1198) strain by addition of dipyridyl, and samples were collected at different time points for RNA extraction. *sodB* and RyhB levels were detected by northern blotting, and both the sRNA and mRNA signals were normalized to the SsrA loading control. Representative blots are shown in (A). *sodB* mRNA decay curves (B) were generated by fitting the normalized signal intensities for each time point in GraphPad Prism. Results represent the mean of at least two independent experiments and bars indicate SEM.

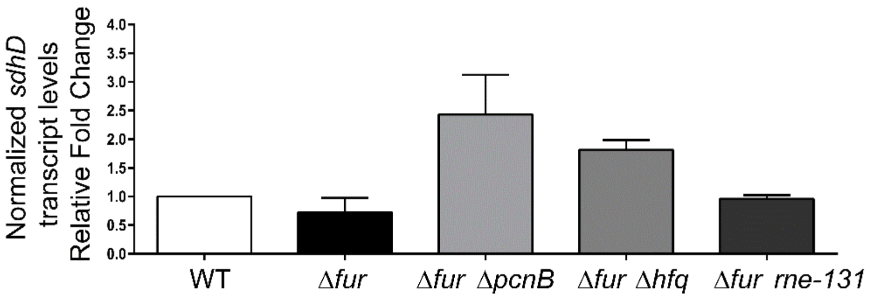

FIGURE S3. Analysis of the effects of deletions in *pcnB*, *hfq* and *rne* on *sdhD* transcript steady-state levels in an *E. coli* Δ*fur* strain. qRT-PCR analysis was used to determine *sdhD* relative transcript levels as described in Materials and Methods. Strains and growth conditions used are described in Figure 3. *sdhD* transcript levels were normalized to that of the internal control (reference gene) SsrA. Results represent the mean of at least three independent experiments and bars indicate SEM.

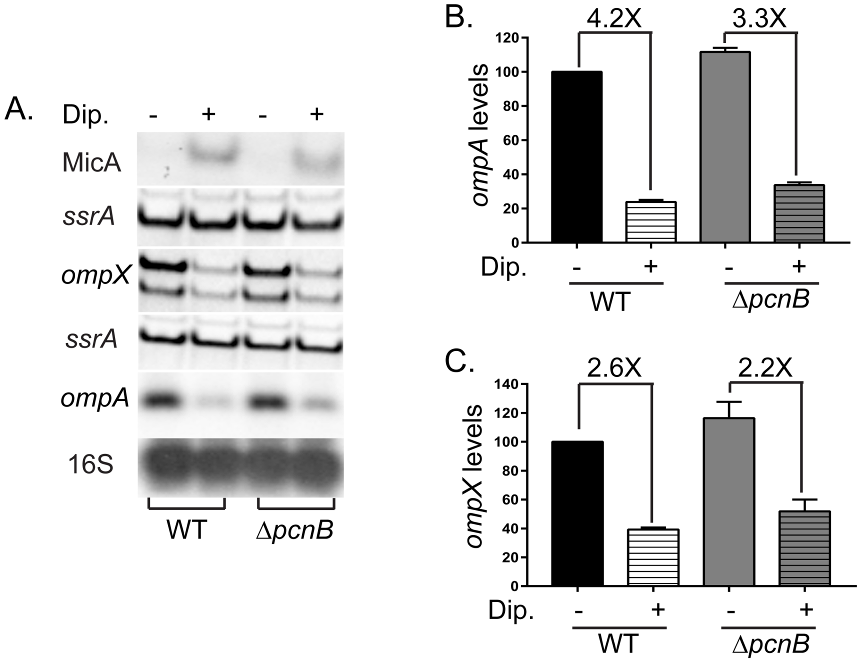

FIGURE S4. Determination of the steady-state levels of MicA targets *ompA* and *ompX* in Δ*pcnB* mutant. Northern blot analysis was performed to determine *ompA* and *ompX* transcript steady-state levels in exponentially growing cultures of a parent (TC279) and an isogenic Δ*pcnB* mutant strain (DS120) under MicA non-inducing (-Dipyridyl) and inducing (+Dipyridyl) conditions; in these strains *micA* is under the control of the *ryhB* promoter. *ompX* and MicA signals were normalized to SsrA loading control while *ompA* signals were normalized to 16S rRNA. Representative northern blots are shown in (A). (B) and (C) show graphs representing the normalized *ompA* and *ompX* mRNA levels, respectively. Results represent the mean of at least three independent experiments and bars indicate SEM. Data analysis was performed as described in the legend of Figure 3B. Probes used are listed in Table S2.

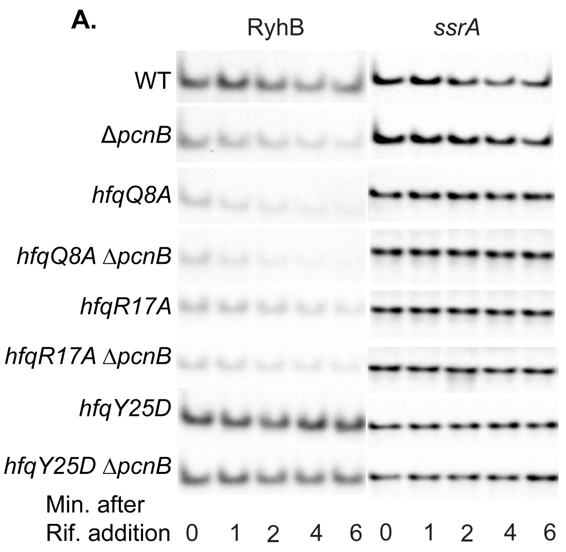

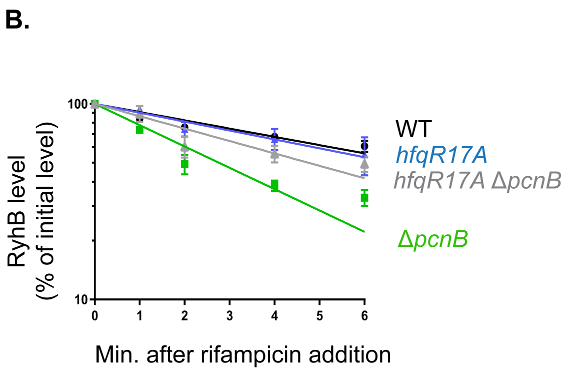

FIGURE S5. Northern blot analysis to determine RyhB stability in the wild-type strain and its derived isogenic *hfq* and *pcnB* mutants. (A) Representative northern blots corresponding to RyhB stability curves shown in Figure 4 F. Northern blot analysis was used to determine RyhB and SsrA (loading control) expression at indicated time points following rifampicin addition in the indicated strain backgrounds under exponential growth conditions. (B) RyhB decay curves corresponding illustrating RyhB stability in the wild-type strain (NRD1138; WT (*fur*$^{+}$)) and its derived mutants (NRD1198; Δ*pcnB*, DS060; *hfqR17A*, and DS073; *hfqR17A* Δ*pcnB*). sRNA decay curves were generated as described in Figure 4 legend and corresponding half-life measurements are listed in Table 1. Points and error bars in the curves represent the means and the standard errors (SEM) of at least three independent experiments.

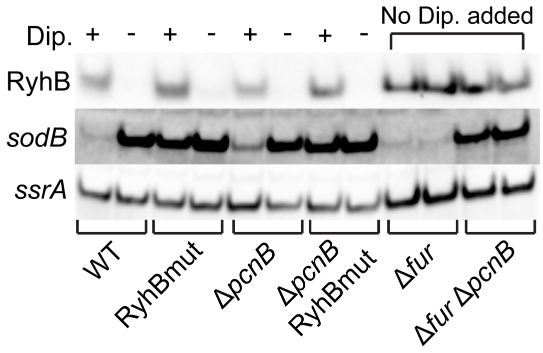

FIGURE S6. Determination of the relative abundance of *sodB* transcript in wild-type (*fur*$^+$; WT) and its derived isogenic *fur* and *pcnB* mutants. Northern blot analysis performed to determine the transcript steady state levels of RyhB target *sodB* under RyhB inducing and non-inducing conditions as described in the legend of Figure 5B in a wild-type (WT (*fur*$^+$); NRD1138) and its derived isogenic mutants (Δ*pcnB*, NRD1198; *ryhBmut*, LM11; *ryhBmut* Δ*pcnB;* Δ*fur,* DS024; Δ*fur* Δ*pcnB*, DS025). Samples for RNA extraction from DS024 and DS025 were collected as described in the legend of Figure 3A. Representative blots showing relative steady-state levels of *sodB*, RyhB, and SsrA in the indicated strain backgrounds are presented. SsrA is used as the loading control.

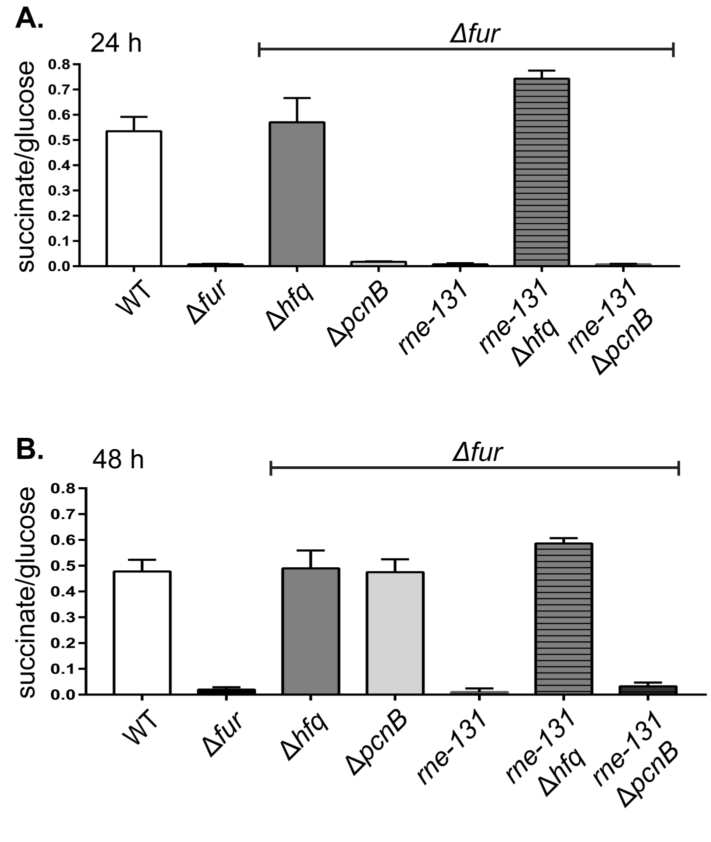

FIGURE S7. The defect in regulation of the *sdhCDAB* mRNA by RyhB in the absence of poly(A) polymerase consequently impacts the ability of *E. coli* to utilize succinate as the sole carbon source. (A, B) Overnight cultures of strain NRD1138 (WT; fur^+), an isogenic Δ*fur* strain (DS024), or derivatives of this Δ*fur* strain harboring additional mutations in *pcnB* (Δ*fur* Δ*pcnB;* DS025)*, hfq* (Δ*fur* Δ*hfq*; DS027) *rne* (Δ*fur rne-131*; DS069), *rne* and *pcnB* (Δ*fur rne-131* Δ*pcnB;* DS082) and, *rne* and *hfq* (Δ*fur rne-131*Δ*hfq*) were grown in M9-glucose media and subcultured in 5 mL of M9-glucose or M9-succinate media to a starting OD_{600} of 0.01. Cultures were subsequently incubated at 37°C with constant shaking. After 24 h and 48 h, growth of each culture was determined by measuring absorbance at 600 nm (OD_{600}). Results represent the mean of the ratio of growth in M9-succinate to M9-glucose of at least three independent experiments for each dataset. Error bars indicate SEM.

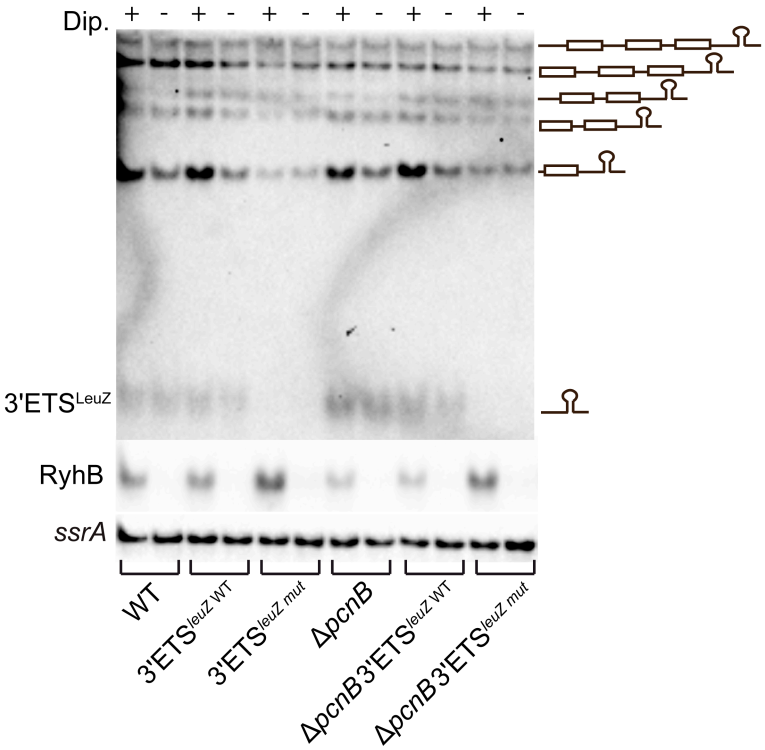

FIGURE S8. Northern blot analysis to determine transcript steady-state levels of LeuZ, 3'ETS^{LeuZ} and RyhB in wild-type and derived isogenic *pcnB* and *leuZ* mutants under RyhB inducing and non-inducing conditions. Representative northern blots corresponding to Figure 6C. Strains and growth conditions used are described in the legend of Figure 6C. Experiment was performed in triplicate and northern blots representing transcript steady-state levels of LeuZ, 3'ETS^{LeuZ} and RyhB are shown. SsrA was used as the loading control. LeuZ term probe (Table S2) was used to determine LeuZ and 3'ETS^{LeuZ} levels.

A.

RyhB	GCGATCAGGAAGACCCTCGCGGAGAACCTGAAAGCACGACATTGCTCACATTGCTTCCAGTATTACTTAGCCAGCCGGGTGCTGGCTTTTTTTTT	95
c18	GCGATCAGGAAGACCCTCGCGGAGAACCTGAAAACACGACATTGCTCACATTGCTTCCAGTATTACTTAGCCAGCCGGGTGCTGGCTTTTT	91
c24	CGATCAGGAAGACCCTCGCGGAGAACCTGAAAGCACGACATTGCTCACATTGCTTCCAGTATTACTTAGCCAGCCGGGTGCTGGCTTTTTTT**A**	93
c29	GCGATCAGGAAGACCCTCGCGGAGAACCTGAAAGCACGACATTGCTCACATTGCTTCCAGTATTACTTAGCCAGCCGGGTGCTGGCTTTTT	91
c32	GCGATCAGGAAGACCCTCGCGGAGAACCTGAAAGCACGACATTGCTCACATTGCTTCCAGTATTACTTAGCCAGCCGGGTGCTGGCTTTTT**A**	92
c33	GCGATCAGGAAGACCCTCGCGGAGAACCTGAAAGCACGACATTGCTCACATTGCTTCCAGTATTACTTAGCCAGCCGGGTGCTGGCTTTTTT**A**	93
c34	GCGATCAGGAAGACCCTCGCGGAGAACCTGAAAGCACGACATTGCTCACATTGCTTCCAGTATTACTTAGCCAGCCGGGTGCTGGCTTTTTTT**A**	94
c46	GCGATCAGGAAGACCCTCGCGGAGAACCTGAAAGCACGACATTGCTCACATTGCTTCCAGTATTACTTAGCCNGCCGGGTGCTGGCTTTTTT**A**	93

(Position 90 is marked by an arrowhead on the RyhB sequence.)

B.

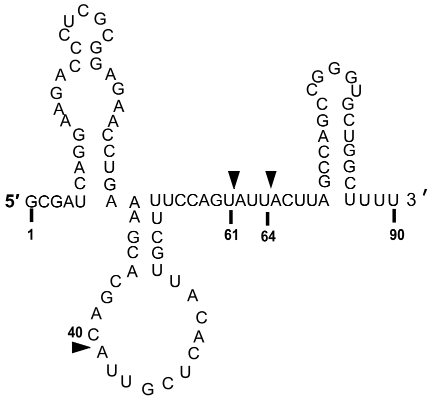

C.

RyhB degradation products from DS024 (Δ*fur)*		RyhB degradation products from NRD1138 (WT)	
**Position of termination**	**Number of clones terminated**	**Position of termination**	**Number of clones terminated**
nt # 61	10	nt # 61	4
nt # 40	7	nt # 40	17
nt # 31	2	nt # 31	2
nt # 58	1	nt # 58	1
nt # 49	1	nt # 49	4
nt # 64	8	nt # 52	2
nt # 21	2	nt # 49	1
nt # 82	1	nt # 60	1
nt # 39	1	nt # 48	1
nt # 28	1	nt # 37	1
		nt # 34	1

FIGURE S9. Analysis of 3′-end polyadenylation state of RyhB. 3′ RACE was used to determine the sequence at the 3' ends of RyhB from exponential cultures of the Δ*fur* mutant and the wild type (WT; control) strain. Full-length RyhB (90 nt.) sequence was detected in 7 out of 47 clones sequenced from the Δ*fur* mutant and 5 out of 7 clones consisted of a template independent terminal adenine at the 3′ end (A). RyhB sequence as annotated in *E. coli* MG1655 is highlighted in red (A). The remaining 40 clones from the Δ*fur* mutant and all clones sequenced from the WT control yielded RyhB degradation products of varying lengths (C) and most RyhB degradation products terminated at nucleotide positions 40, 61 and 64 (B).

SUPPLEMENTAL MATERIALS AND METHODS

Strain construction

Strains generated by P1 *vir* transduction are indicated in Supplementary Table S1 with the donor strain indicated in brackets, using the protocol described by Miller (Miller 1992) and appropriate antibiotic selection.

Strain NRD698: Electrocompetent lambda Red induced NM600T cells were transformed with DNA generated from PCR amplification of the chloramphenicol resistance cassette from pKD3 using *pcnB* KO For and Rev primers. Successful recombinants were isolated on LB plates containing chloramphenicol and were validated by colony PCR using *pcnB* for and rev primers.

Strain NRD1038: Lambda Red induced NM600T cells were transformed via electroporation with a PCR product generated from amplification of the *kan*-pBAD-*ccdB* cassette from NM570 with Taq DNA polymerase using RyhBccdBkan For and Rev primers. Recombinants were selected for on LB plates containing kanamycin, then screened for arabinose sensitivity and tetracycline resistance (maintenance of mini-λ). Successful recombinants that were kanamycin and tetracycline resistant, but arabinose sensitive were validated by colony PCR using RyhBKOchk For and Rev primers.

Strain NRD1362: Electrocompetent lambda red induced KR10000 harboring a mini-lambda phage were transformed with DNA generated from PCR amplification of the zeocin resistance cassette was amplified from NRD676 genomic DNA using *fur::zeo* For and Rev primers. Successful recombinants were isolated on LB plates containing zeocin and validated by colony PCR using furKOchk For and Rev primers.

NRD1530: Lambda Red induced NM1200 cells were transformed via electroporation with a PCR product generated by amplification of a tetracycline resistance cassette from NRD654 with Taq DNA polymerase using asnBKOtet For and Rev primers. Cells were subsequently isolated

on LB plates containing tetracycline and validated by colony PCR using asnBKOtetchk For and Rev primers.

NRD1576: A kanamycin resistance cassette was inserted immediately after the Rho-independent terminator encoded downstream of *leuZ* by transforming electrocompetent Lambda Red recombinase induced KR10000 cells with a PCR product created by amplification of the kanamycin resistance cassette from pKD4 with primers leuZwtKOin For and leuZKOin Rev using Taq DNA polymerase. Successful recombinants were selected on LB plates containing kanamycin and validated by colony PCR using leuZKOchk For and Rev primers.

NRD1578: Four point mutations were introduced into the sequence encoding the $3'ETS^{LeuZ}$ and a *kan* cassette was simultaneously inserted downstream of its encoded Rho-independent terminator by Lambda Red-mediated recombineering. Electrocompetent Lambda Red induced KR10000 cells were transformed with a PCR product generated from the template plasmid pKD4 with Taq DNA polymerase using primers leuZdblinvKOin For and leuZKOin Rev. Successful recombinants were selected on LB plates containing kanamycin and validated by sequencing of the DNA produced from colony PCR using leuZKOchk For and Rev primers.

LM06: Strain NRD1038λ harboring mini-lambda phage and the Kn-pBAD-*ccdB* cassette was transformed with the RyhBmut gBlock (IDT, Table S2) containing the mutated RyhB sequence with 40 bp of homology on either side of RyhB. Successful recombinants ($Ara^R Kan^S$) were sequence verified for the RyhB mutation after colony PCR amplification using RyhBKOchk forward and reverse primers.

Succinate growth assay

Strains were initially grown on M9-glucose agar plates and single colonies for each strain was inoculated in 2 mL of M9-glucose broth and grown overnight at 37^oC aerobically.

Each overnight culture was subcultured separately into 5 mL of fresh M9-glucose broth and M9-succinate broth to a starting OD_{600} of 0.01. Cultures were grown aerobically at 37°C and growth was determined by measuring OD_{600} after 24 h and 48 h. Final growth yield in succinate at the end of each time point was expressed as a ratio of the OD_{600} obtained for growth in M9-succinate to that in M9-glucose corresponding to each strain culture.

Northern blot analysis of *ompA*

Northern blot analysis of *ompA* was carried out as described previously (De Lay and Gottesman 2009). Briefly, 8 µg of each RNA sample was loaded on a 1.2% agarose gel that was pre-run at 12V/cm for at least 5 min and subsequently run at 5V/cm for 2 h in 1X MOPS (morpholinepropanesulfonic acid) buffer. Next, the RNA samples were transferred to a Zeta-Probe GT membrane (Bio-Rad) via capillary transfer. Transferred RNA was UV crosslinked and hybridized overnight with 100 ng/mL of 5′ biotinylated OmpA probe (Supplemental Table S2) as described in the Materials and Methods. Signal intensity corresponding to *ompA* was normalized to that of 16S rRNA, which served as internal loading control.

3′ RACE

3′-RACE assays were carried out as described previously (Argaman et al. 2001) with minor modifications. Total RNA was isolated from exponential phase cultures growing in MOPS EZ rich defined media as described in Materials and Methods section and was subjected to DNase treatment (DNase Turbo; Ambion) according to manufacturer’s guidelines. 15 µg of DNase-treated RNA was dephosphorylated using calf intestine alkaline phosphatase (CIP; NEB). 3′ RNA adapter (E1, 5′-phosphate UUCACUGUUCUUAGCGGCCGCAUGCUC-idT- 3′) ligation was performed overnight (16 h) at 37°C with T4 Ligase (NEB). Reverse transcription of

the ligated RNA samples was carried out as described in Materials and Methods section, but with 100 pmol of E1 DNA adapter (primer complementary to E1 RNA adapter (Table S2)). The products of RT-reactions (2 μL) were PCR amplified with Taq DNA polymerase (NEB) using the following primer pairs - RyhB 3′RACE and E1 DNA adapter (Table S2). PCR products were size checked and cloned into pCR 2.1 TOPO-vector (Invitrogen) following manufacturer's protocol. Bacterial clones obtained after transformation were assayed for the presence of inserts of expected size by colony PCR using primer pairs M13 For and M13 Rev (Table S2). Plasmids from clones containing appropriate sized inserts were purified using QIAprep Spin miniprep kit (Qiagen) and sequenced with M13 For primer.

SUPPLEMENTAL REFERENCES

Argaman L, Hershberg R, Vogel J, Bejerano G, Wagner EG, Margalit H, Altuvia S. 2001. Novel small RNA-encoding genes in the intergenic regions of *Escherichia coli*. *Curr Biol* **11**: 941-950.

Baba T, Ara T, Hasegawa M, Takai Y, Okumura Y, Baba M, Datsenko KA, Tomita M, Wanner BL, Mori H. 2006. Construction of *Escherichia coli* K-12 in-frame, single-gene knockout mutants: the Keio collection. *Mol Syst Biol* **2**: 2006 0008.

Bandyra KJ, Sinha D, Syrjanen J, Luisi BF, De Lay NR. 2016. The ribonuclease polynucleotide phosphorylase can interact with small regulatory RNAs in both protective and degradative modes. *RNA* **22**: 360-372.

Blattner FR, Plunkett G, 3rd, Bloch CA, Perna NT, Burland V, Riley M, Collado-Vides J, Glasner JD, Rode CK, Mayhew GF et al. 1997. The complete genome sequence of *Escherichia coli* K-12. *Science* **277**: 1453-1462.

Cameron TA, De Lay NR. 2016. The Phosphorolytic Exoribonucleases Polynucleotide Phosphorylase and RNase PH Stabilize sRNAs and Facilitate Regulation of Their mRNA Targets. *J Bacteriol* **198**: 3309-3317.

Datsenko KA, Wanner BL. 2000. One-step inactivation of chromosomal genes in *Escherichia coli* K-12 using PCR products. *Proc Natl Acad Sci U S A* **97**: 6640-6645.

De Lay N, Gottesman S. 2009. The Crp-Activated Small Noncoding Regulatory RNA CyaR (RyeE) links Nutritional Status to Group behaviour. *J Bacteriol* **191**: 461-476.

De Lay N, Gottesman S. 2011. Role of polynucleotide phosphorylase in sRNA function in *Escherichia coli*. *RNA* **17**: 1172-1189.

Guillier M, Gottesman S. 2008. The 5' end of two redundant sRNAs is involved in the regulation of multiple targets, including their own regulator. *Nucleic Acids Res* **36**: 6781-6794.

Masse E, Escorcia FE, Gottesman S. 2003. Coupled degradation of a small regulatory RNA and its mRNA targets in *Escherichia coli*. *Genes Dev* **17**: 2374-2383.

Miller JH. 1992. *A Short Course in Bacterial Genetics*. Cold Spring Harbor Laboratory Press, Cold Spring Harbor, NY.

Schu DJ, Zhang A, Gottesman S, Storz G. 2015. Alternative Hfq-sRNA interaction modes dictate alternative mRNA recognition. *EMBO J* **34**: 2557-2573.
Zhang A, Wassarman KM, Ortega J, Steven AC, Storz G. 2002. The Sm-like Hfq protein increases OxyS RNA interaction with target mRNAs. *Mol Cell* **9**: 11-22.
Zhang A, Wassarman KM, Rosenow C, Tjaden BC, Storz G, Gottesman S. 2003. Global analysis of small RNA and mRNA targets of Hfq. *Mol. Microbiol* **50**: 1111-1124.
